# Development of novel fluorescent histamine H_1_-receptor antagonists to study ligand-binding kinetics in living cells

**DOI:** 10.1038/s41598-018-19714-2

**Published:** 2018-01-25

**Authors:** Leigh A. Stoddart, Andrea J. Vernall, Monica Bouzo-Lorenzo, Reggie Bosma, Albert J. Kooistra, Chris de Graaf, Henry F. Vischer, Rob Leurs, Stephen J. Briddon, Barrie Kellam, Stephen J. Hill

**Affiliations:** 1 Division of Pharmacology Physiology and Neuroscience, School of Life Sciences, University of Nottingham, Nottingham, NG7 2UH UK; 2 School of Pharmacy, Division of Biomolecular Science and Medicinal Chemistry, Centre for Biomolecular Sciences, University of Nottingham, Nottingham, NG7 2RD UK; 3 Department of Chemistry and Pharmaceutical Sciences, Division of Medicinal Chemistry, Amsterdam Institute for Molecules, Medicines and Systems (AIMMS), Vrije Universiteit, Amsterdam, PO Box 7161 Amsterdam, The Netherlands; 4 Centre of Membrane Proteins and Receptors, University of Birmingham and University of Nottingham, Midlands, UK

## Abstract

The histamine H_1_-receptor (H_1_R) is an important mediator of allergy and inflammation. H_1_R antagonists have particular clinical utility in allergic rhinitis and urticaria. Here we have developed six novel fluorescent probes for this receptor that are very effective for high resolution confocal imaging, alongside bioluminescence resonance energy transfer approaches to monitor H_1_R ligand binding kinetics in living cells. The latter technology exploits the opportunities provided by the recently described bright bioluminescent protein NanoLuc when it is fused to the N-terminus of a receptor. Two different pharmacophores (mepyramine or the fragment VUF13816) were used to generate fluorescent H_1_R antagonists conjugated via peptide linkers to the fluorophore BODIPY630/650. Kinetic properties of the probes showed wide variation, with the VUF13816 analogues having much longer H_1_R residence times relative to their mepyramine-based counterparts. The kinetics of these fluorescent ligands could also be monitored in membrane preparations providing new opportunities for future drug discovery applications.

## Introduction

G protein-coupled receptors (GPCR) are one of the major targets for currently approved drugs, with *circa* 30% acting at the GPCR superfamily^1^. Furthermore, there remains huge potential for innovation within this protein family since only 30% of the non-olfactory GPCRs have been successfully targeted^2^. The development of new therapeutics has been hampered in recent years, however, by the failure of many drugs in late-stage clinical trials as a consequence of a lack of appropriate clinical efficacy^3^. The increasing number of crystal structures available for GPCRs has facilitated the application of rational design efforts to the drug discovery process^4,5^ but since these receptors are highly dynamic proteins that can adopt a wide range of conformations, there is a need to study these receptors in their natural cellular environment^6^.

An important, but often overlooked, property of a drug candidate is the rate at which it binds to, and dissociates from, its target receptor^7^. Drugs with similar affinity can display markedly different binding kinetics, and optimising a drug’s binding kinetics to clinical need is thought to be one way to reduce drug discovery attrition rates^8,9^. The use of isolated membranes from homogenized cells in combination with radiolabelled ligands has been the most frequently used method to measure ligand binding kinetics to a GPCR. However, intracellular signalling proteins can have marked allosteric influences on the binding of ligands to GPCRs^10–12^ and one consequence of allosteric interactions is that they change ligand binding kinetics^13^. As a result of this, there may be differences in the binding kinetics of compounds measured in whole cells compared to those measurements made in isolated membranes. One way to study ligand-binding kinetics of receptors in their natural cellular environment is through the use of fluorescently labelled agonists and antagonists^14,15^.

Fluorescent ligands for GPCRs have been used to study various aspects of receptor pharmacology and function including ligand binding^16,17^, endogenous receptor localisation^18–20^, receptor organisation within the cell membrane^21,22^ and ligand binding kinetics^23–25^. However, fluorescent ligands often require optimisation for use in a specific application. For example, in the case of the histamine H_1_ receptor (H_1_R), Rose *et al*. described a fluorescent ligand that was successfully used for fluorescence correlation spectroscopy, but showed high levels of non-specific membrane binding and cytoplasmic uptake that made confocal imaging difficult^26^. Fluorescent ligands for GPCRs are normally composed of an agonist or antagonist for the receptor of interest chemically linked to a fluorescent molecule such as one of the boron-dipyrromethene (BODIPY) or cyanine dyes^27^. For fluorescent small molecule ligands, such as those for Class A GPCRs, addition of the linker and fluorophore results in a significant increase in molecular weight and complexity and these molecules should therefore be treated as new pharmacological entities. For instance, we have shown that the linker region can influence the fluorescent ligand properties, with peptide-based linkers improving both affinity and imaging capabilities of Xanthine Amine Congener based ligands for the adenosine A_3_ receptor^28^.

The H_1_R is a Class A GPCR which couples predominantly to G_q/11_ proteins, leading to phospholipase C activation and release of intracellular calcium^29^. It is expressed in a wide variety of cell types including, smooth muscle, endothelium, immune cells and neurons^30^, and its activation by the endogenous ligand histamine is a key mediator of allergy and inflammation. A wide range of clinically approved antagonists for the H_1_R have been successfully used for many years in the treatment of allergy related conditions such as allergic rhinitis and urticaria^31^. The crystal structure of an antagonist bound H_1_R has been recently solved^32^ and therefore to gain further understanding of the dynamic regulation of ligand binding to this receptor in live cells, improvements to the currently available fluorescent ligands^26^ needed to be made. By using the strategy previously described for the adenosine A_3_ receptor^28^, where peptide linkers were introduced between the fluorophore and pharmacophore, we aimed to generate broad utility fluorescent ligands for the H_1_R that are suitable for use in a range of different fluorescent techniques including confocal microscopy, but particularly for assessing receptor binding kinetics using bioluminescence resonance energy transfer (BRET).

## Results

### Pharmacological Characterization of Fluorescent Ligands

**Figure 1:**
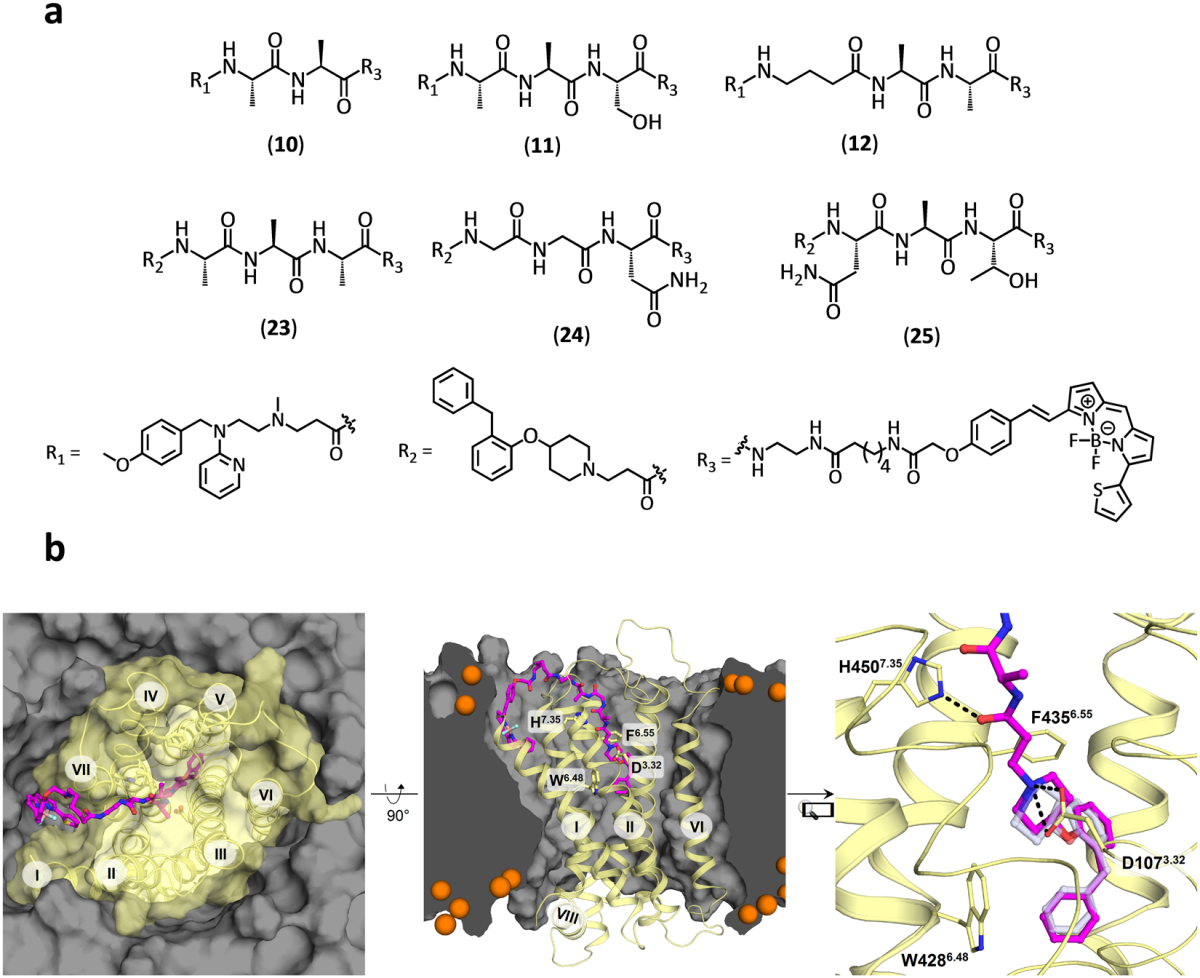
Chemical structure and molecular modelling of fluorescent ligands for the H_1_R. (**a**) Chemical structures of fluorescent mepyramine (**10, 11, 12**) and VUF13816 (**23, 24, 25**) derivatives (**b**) Modelled binding pose of **23** in the H_1_R, based on the experimentally validated binding mode of VUF13816^34,35^ in the H_1_R crystal structure (PDB code: 3RZE)^32^.

Six fluorescent antagonists for the H_1_R were synthesized as part of this study (Figure S1); three based on the commonly used H_1_R antagonist mepyramine and three based on the recently described fragment-like antagonist VUF13816^33,34^ (Fig. 1a). The di- or tripeptide linkers were designed based on the predicted docking pose of VUF13816^34^ in the H_1_R crystal structure^32^ (Fig. 1b). The binding mode of VUF13816 has been experimentally validated by combined structure-activity relationship and site-directed mutagenesis studies^35^ and suggests that the basic amine nitrogen atom that interacts with the anionic D107^3.32^ residue in TM helix III provides a good point to attach a peptide linker that: i) has the appropriate length to cross the extracellular vestibule to place the fluorophore in the membrane bilayer, and ii) is compatible with the H_1_R binding surface (Fig. 1b). 6-(((4,4-Difluoro-5-(2-thienyl)-4-bora-3*a*,4*a*-diaza-*s*-indacene-3-yl)styryloxy)acetyl)aminohexanoic acid (BODIPY630/650-X) was preferred due to previous success with this fluorophore for Class A GPCR fluorescent ligands^28,36,37^ as it has favourable excitation/emission wavelength for planned techniques. BODIPY630/650 is known to have an increased quantum yield in a lipid environment^38^ and as the modelling suggests the fluorophore would reside within the membrane bilayer this would increase its brightness for imaging studies and reduce background fluorescence in aqueous solution.

**Figure 2:**
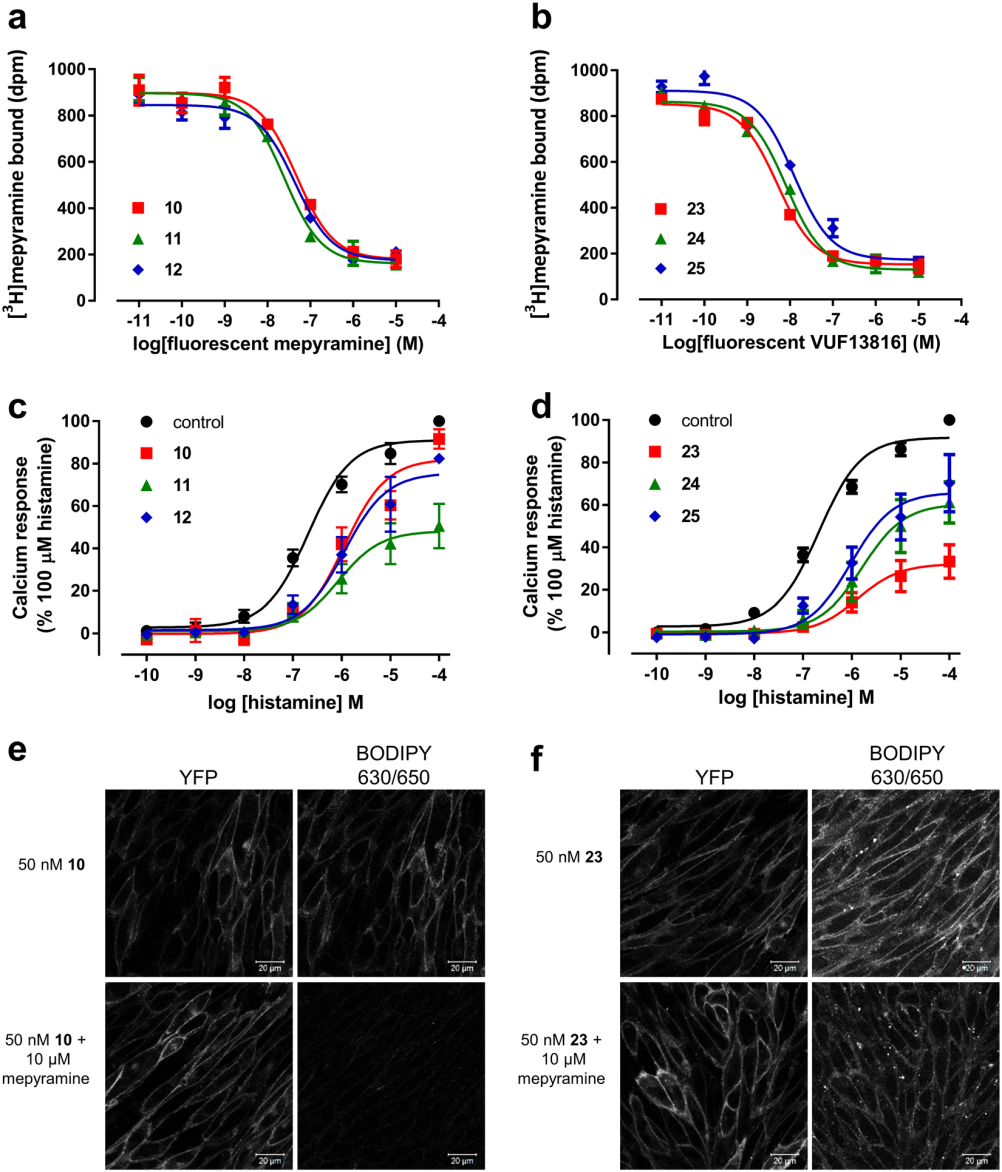
Pharmacological characterisation and live cell confocal imaging of fluorescent ligands at the H_1_R. (**a** and **b**) Inhibition of specific [^3^H]mepyramine binding to cell homogenates transiently expressing the human H_1_R by mepyramine (**a**) and VUF13816 (**b**) based fluorescent antagonists. Data points represent mean ± SEM of triplicate determinations. Graphs shown are representative of three experiments performed. (**c** and **d**) CHO-K1 cells stably expressing the human H_1_R were preincubated with 100 nM of the indicated fluorescent antagonist for 30 min at 37 °C prior to the addition of increasing concentrations of histamine and changes in intracellular calcium monitored. Data were normalized to basal (in the absence of histamine or antagonist) and 100 µM histamine for each experiment. The data shown represent the mean ± SEM of four experiments performed in triplicate. (**e** and **f**) Live cell confocal images of CHO cells expressing H_1_-YFP incubated with 50 nM (**e**) **10** or (**f**) **23** at 37 °C in the absence (top panels) or presence (bottom panels) of 10 µM mepyramine. Single equatorial images were taken of YFP (left hand panels) and BY630/650 (right hand panels). YFP and BY630/650 images are shown in greyscale to avoid issues with colour rendering. For each compound, images in the presence and absence of mepyramine were obtained using identical settings for laser power, detector offset and gain. Data shown are representative of images obtained in three independent experiments. Scale bars = 20 µm.

To confirm that the newly synthesized fluorescent ligands retained the ability to bind to the human H_1_R in HEK293T cells, competition radioligand binding studies were performed. All six compounds retained affinity at the H_1_R (Fig. 2, Table 1) demonstrating that the peptide linker and appended fluorophore were well tolerated and did not lead to a reduction in affinity compared to the parent pharmacophores. No significant difference in the affinity of the mepyramine-based compounds (**10**–**12**) was observed despite the different peptide linkers, with all three compounds displaying affinities in the 10–25 nM range. This was also true for the VUF13816-based compounds (**23**–**25**), although they exhibited slightly higher affinity (4–6 nM) than those based on mepyramine. Each of the compounds was also tested for their ability to antagonize histamine-stimulated calcium release in CHO cells expressing the H_1_R (Fig. 2). All six compounds produced a rightward-shift in the histamine mediated concentration response curves. As seen previously for the H_1_R, a suppression of the maximal calcium response was observed in the presence of each of the compounds, which is related to the non-equilibrium kinetics of the assay^26,39^. Taking this into account EC_25_, rather than EC_50_, values were used to estimate the pK_B_ of the fluorescent compounds (Table 1). The values obtained in the calcium assay were in close agreement with those from the radioligand binding experiments, with the VUF13816 based conjugates again displaying higher affinity than the mepyramine based compounds.

**Table 1:** Binding affinities and kinetic parameters of fluorescent antagonists at the human H_1_R.

Compound	Radioligand binding		Calcium assay		NanoBRET saturation		NanoBRET kinetics				
	pK_i_	*n*	pK_D_	*n*	pK_D_	*n*	k_on_ (× 10^6^ M^−1^min^−1^)	k_off_ (min^−1^)	Tr (min)	pK_D_	*n*
**10**	7.6 ± 0.1	3	7.5 ± 0.2	4	8.1 ± 0.1	6	46.4 ± 5.9	0.214 ± 0.029	4.99 ± 0.78	8.3 ± 0.01	4
**11**	8.0 ± 0.1	3	7.9 ± 0.1	4	7.2 ± 0.1	4	19.8 ± 2.2	0.126 ± 0.015	8.40 ± 0.91	8.2 ± 0.06	5
**12**	7.7 ± 0.1	3	7.8 ± 0.1	4	7.3 ± 0.2	5	3.3 ± 0.6	0.134 ± 0.011	7.68 ± 0.58	7.4 ± 0.09	6
**23**	8.4 ± 0.1	3	8.6 ± 0.2	4	8.1 ± 0.1	6	1.6 ± 0.3	0.019 ± 0.002	54.08 ± 5.33	7.9 ± 0.10	4
**24**	8.3 ± 0.1	3	8.1 ± 0.3	4	7.6 ± 0.1	5	2.9 ± 0.6	0.027 ± 0.002	37.81 ± 3.44	8.0 ± 0.09	4
**25**	8.2 ± 0.2	3	7.8 ± 0.3	4	7.5 ± 0.1	5	2.6 ± 0.4	0.030 ± 0.006	39.53 ± 9.40	8.0 ± 0.10	4

pK_i_ values were calculated from inhibition of [^3^H]mepyramine binding to membranes from HEK293T cells transiently expressing human H_1_R. Calcium assay pK_D_ values were estimated from a shift in histamine concentration response curves to 100 nM of each fluorescent compound in CHO cells expressing H_1_R. NanoBRET saturation pK_D_ values were calculated from the saturation curve of the fluorescent ligands binding to HEK293T cells expressing Nluc-H_1_R. The kinetic parameters, k_on_, k_off_ and pK_D_ values were obtained using varying concentrations of fluorescent ligand and measuring the NanoBRET signal over time in HEK293T cells expressing Nluc-H_1_R. The non-specific signal was determined using a high concentration (10 μM) of doxepin as a competitor. The residence time (Tr) was calculated as the mean of the reciprocal of the k_off_ values from each individual experiment. All values represent mean ± SEM from *n* separate experiments performed in triplicate.

### Confocal Microscopy

Peptide linkers were used between the pharmacophore and fluorophore component of the fluorescent ligands in an attempt to reduce the compounds’ lipophilicities and ability to cross cell membranes (compared to the equivalent alkyl linker), thus optimising their properties for use in confocal imaging. Imaging studies were performed on CHO cells expressing H_1_R linked to yellow fluorescent protein (H_1_-YFP), which was predominantly expressed at the cell surface. Exposure of H_1_-YFP cells to 50 nM of **10**, **11** or **12** for 30 min at 37 °C resulted in clear membrane localisation of the BODIPY630/650 fluorescence emission for each of the ligands (Fig. 2 and Figure S2). To confirm the specificity of binding to the H_1_R, H_1_-YFP expressing cells were pre-treated with 10 µM mepyramine prior to the addition of the fluorescent mepyramine derivatives and subsequent imaging. Under these conditions, very little fluorescence was observed for **10** and **12**. However, some residual cell surface fluorescence was observed for **11**. To illustrate the improvement in the imaging properties obtained with the peptide linkers, cells were also exposed to a derivative with a non-peptidic linker, mepyramine-X-BODIPY630/650^26^, in the presence and absence of mepyramine. In contrast to the ligands with peptidic linkers, very little cell surface binding of mepyramine-X-BODIPY630/650 could be discerned due to high levels of intracellular accumulation of the fluorescent ligand. This was not prevented by the presence of mepyramine, indicating significant non-specific binding and cellular uptake (Figure S3). The three VUF13816-based compounds (**23**–**25**) were also imaged in the presence and absence of mepyramine (Fig. 2 and S3). These fluorescent compounds also displayed clear cell surface binding which showed a similar binding pattern to the H_1_R-YFP fluorescence. For each of the compounds clear reduction in cell surface fluorescence was observed following pre-treatment with the unlabelled antagonist mepyramine indicating the majority of the membrane fluorescence was specific binding to the H_1_R. Some residual binding was observed with **23**, which may be due to it being the highest affinity of the fluorescent ligands tested.

### Saturation and competition binding studies using NanoBRET in whole cells

**Figure 3:**
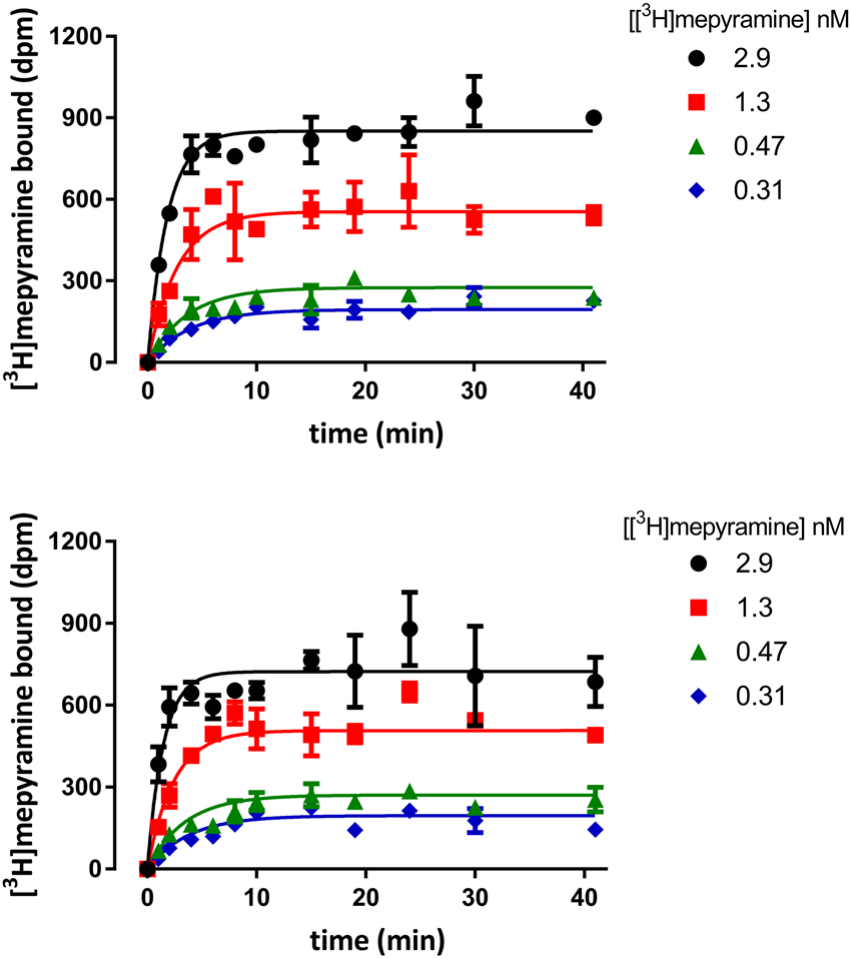
Association binding of [^3^H]mepyramine at HA-H_1_R and Nluc-H_1_R. Specific [^3^H]mepyramine binding over time to cell homogenate transiently expressing the human H_1_R was measured for cells expressing the HA-tagged receptor (**a**) or the Nluc-tagged receptor (**b**). Data points represent mean ± SEM of duplicate determinations. Graphs shown are representative of 12 (a) or 4 (b) experiments performed.

**Figure 4:**
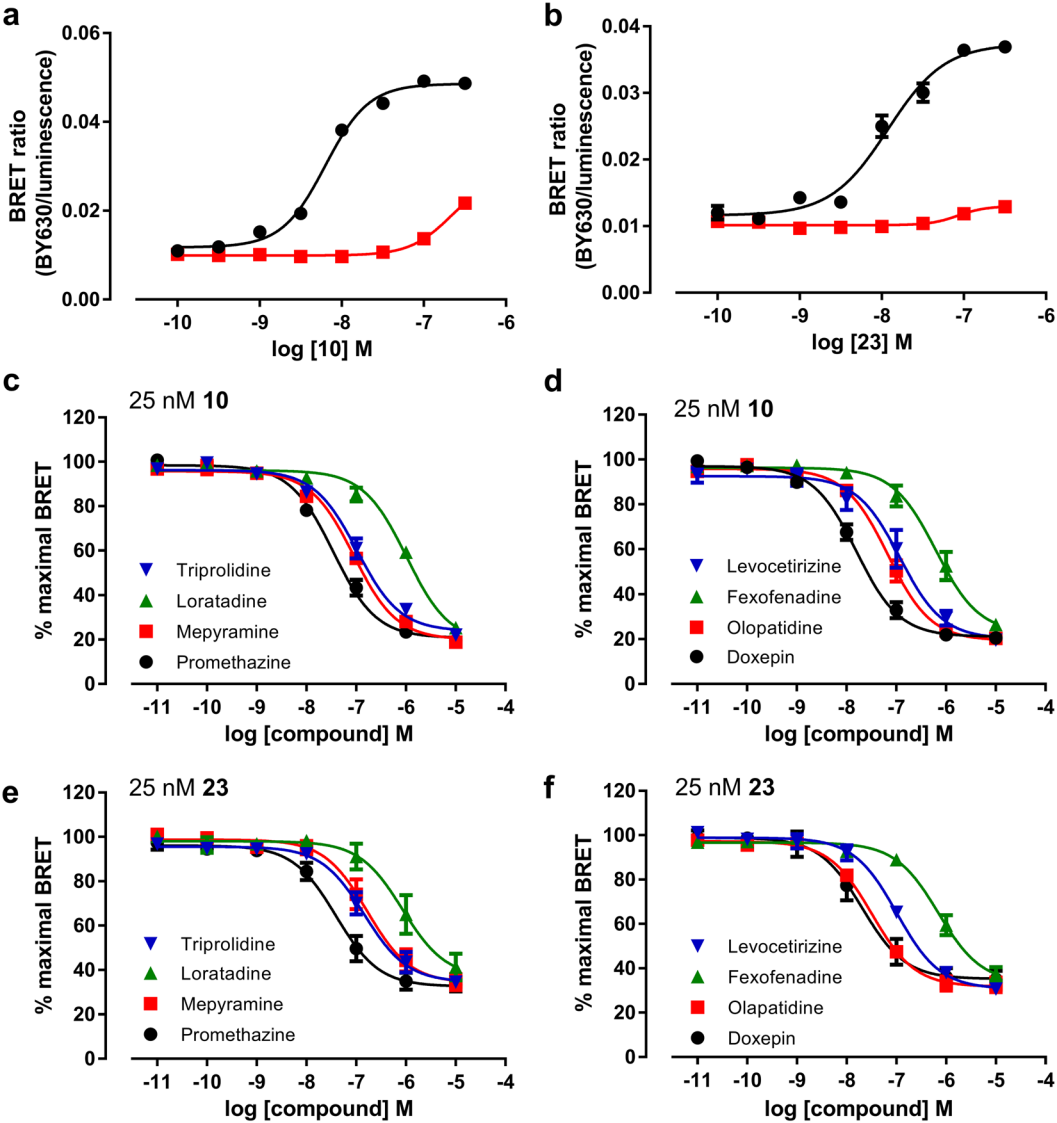
Saturation and competition NanoBRET studies with Fluorescent Ligands 10 and 23. (**a** and **b**) Saturation binding curves from BRET experiments in Nluc-H_1_R HEK cells treated with increasing concentrations of (**a**) **10** or (**b**) **23** in the absence (black circles) or presence (red squares) of 10 µM mepyramine. The data shown are representative of six independent experiments performed in triplicate. (**c**–**f**) Inhibition of BRET signal in Nluc-H_1_R HEK293T cells treated with 25 nM **10** (**c,d**) or 25 nM **23** (**e**,**f**) and increasing concentrations of the unlabelled ligands; tripolidine, loratadine, mepyramine and promethazine (**c**,**e**) or levocetirizine, fexofenadine, olopatadine and doxepin (**d**,**f**). Data were normalized to maximal BRET signal in the absence of competitor. The data shown represent the mean ± SEM of at least three experiments (as detailed in Table 3) performed in triplicate.

Bioluminescence resonance energy transfer (BRET) is a proximity (<10 nm) assay which can be used to measure the binding of fluorescent ligands to a receptor of interest expressing a bioluminescent protein (NanoLuc) tag on the N-terminus^40,41^. We used our fluorescent ligands in conjunction with a NanoLuc tagged H_1_R (Nluc-H_1_R) to determine their binding kinetics at the receptor. Initially, to confirm that the NanoLuc tag had no steric hindrance effects on the binding of ligands to the H_1_R, we performed association binding experiments using [^3^H]mepyramine to determine its kinetic parameters at both HA-H_1_R and Nluc-H_1_R (Fig. 3). The association and dissociation rates of [^3^H]mepyramine at Nluc-H_1_R and HA-H_1_R, and consequently its affinity, were not significantly different (Table 2). All six of the fluorescent ligands used in this study showed a clear concentration-dependent and saturable increase in BRET signal when incubated with HEK293T cells stably expressing Nluc-H_1_ (Fig. 4 and S4). Low levels of non-specific BRET were observed for all fluorescent ligands, as determined by co-incubation with a high concentration (10 µM) of unlabelled mepyramine. Due to the wide concentration range of fluorescent ligands used in these studies, specific binding was plotted versus ligand concentration on a log scale and fitted with a sigmoidal function to determine the K_D_ value as the concentration at which 50% specific binding is achieved. In addition, the wide concentration range of fluorescent ligand used also appeared to cause an increase in the non-specific binding at the highest concentration of fluorescent ligand used (300 nM). This was almost certainly due to a direct competition of fluorescent and unlabelled ligand for the receptor with the extent of the shift in the log concentration-binding curve effectively reflecting the affinity of mepyramine for the H_1_R. The K_D_ values obtained for each of the six fluorescent H_1_R antagonists in Nluc-H_1_R-HEK293T cells are shown in Table 1.

**Table 2:** Kinetic parameters of [^3^H]mepyramine at HA-H_1_R and Nluc-H_1_R.

	k_on_ (×10^6^ M^−1^ min^−1^)	k_off_ (min^−1^)	pK_D_	*n*
HA-H_1_R	122 ± 7	0.21 ± 0.01	8.7 ± 0.0	12
Nluc-H_1_R	147 ± 16	0.24 ± 0.02	8.8 ± 0.0	4

Kinetic parameters for [^3^H]mepyramine-receptor binding were determined by monitoring [^3^H]mepyramine binding to a homogenate of HEK293T cells expressing either HA-H_1_R or Nluc-H_1_R. All values represent mean ± SEM from *n* separate experiments performed in duplicate.

To further study the suitability of these novel fluorescent ligands to monitor ligand binding at the H_1_R we used the mepyramine- and VUF13816-based fluorescent ligands with the highest affinity for the receptor (**10** and **23**) to perform competition binding assays (Fig. 4). All eight compounds caused a concentration-dependent decrease in BRET ratio with both fluorescent ligands and this allowed pK_i_ values of eight diverse H_1_R antagonists to be determined (Table 3). In general, the pK_i_ values obtained using **10** or **23** were comparable (R^2^ = 0.91) with doxepin displaying the highest affinity for the H_1_R and loratadine the lowest. Of the eight compounds tested, two compounds showed a small degree of probe-dependence; mepyramine was found to have a slightly higher affinity when using **10** as the tracer whereas olopatadine was slightly higher affinity in competition with **23** (p < 0.05, unpaired t-test).

**Table 3:** Affinities of unlabelled antagonists at Nluc-H_1_R measured in whole cells and membranes.

Compound	25 nM **10**				25 nM **23**	
	Whole cells		Membranes		Whole cells	
	pK_i_	*n*	pK_i_	*n*	pK_i_	*n*
Promethazine	8.12 ± 0.02^†^	5	8.67 ± 0.07^†^	4	7.94 ± 0.12	4
Mepyramine	7.62 ± 0.05^*^	5	7.77 ± 0.14	4	7.28 ± 0.12^*^	5
Loratadine	6.48 ± 0.05^†^	5	6.21 ± 0.09^†^	4	6.36 ± 0.10	4
Triprolidine	7.55 ± 0.11	4	7.77 ± 0.09	4	7.34 ± 0.13	5
Doxepin	8.36 ± 0.10^†^	5	9.06 ± 0.15^†^	4	8.30 ± 0.13	5
Olopatadine	7.73 ± 0.10^*†^	5	8.40 ± 0.13^†^	4	8.05 ± 0.04^*^	4
Fexofenadine	6.78 ± 0.18	5	7.00 ± 0.11	4	6.70 ± 0.10	4
Levocetirizine	7.28 ± 0.06^†^	3	7.66 ± 0.08^†^	4	7.38 ± 0.10	5

pK_i_ values of eight H_1_R antagonists in HEK293T cells or membranes expressing Nluc-H_1_R were determined by competition binding with 25 nM **10** (whole cells and membranes) or 25 nM **23** (whole cells only). All values represent mean ± SEM from *n* separate experiments performed in triplicate. ^*^p < 0.05 pK_i_ measured using **10** versus **23** (Student’s unpaired *t* test). ^†^p < 0.05 pK_i_ measured using **10** in whole cells versus membranes (Student’s unpaired *t* test).

### Suitability of NanoBRET for kinetic studies

The saturation and competition measurements described above were performed as end point readings with the assumption that equilibrium had been reached. If this was correct, then the pK_i_ values obtained should be independent of the kinetics of the fluorescent and non-fluorescent compounds used. To test this, we first used the NanoBRET assay to directly determine the kinetic constants (k_on_ and k_off_) of the fluorescent ligands. As the BRET measurements are dependent on the luminescence from the NanoLuc protein, and since kinetic measurements may need to be taken for up to 2 hours, it was necessary to confirm that the BRET signal was stable over this time. To test this, Nluc-H_1_R HEK293T cells were treated with 25 nM **10** in the presence and absence of 10 μM doxepin for 60 min prior to the addition of 0.5 µM furimazine (Nluc substrate). Luminescence and fluorescence emissions were monitored every minute for 90 min and then an additional 0.5 µM furimazine was added and luminescence and fluorescence monitored for a further 30 min. As seen in Figure S5, fluorescence and luminescence signals decayed with time after the addition of the substrate (due to consumption of the substrate by the bioluminescence reaction) and exhibited an instant increase after the addition of additional furimazine which subsequently decayed again with time. Due to the ratiometric nature of the BRET signal, however, the resulting BRET ratio was found to be very stable 15 min following the addition of substrate, even after the second substrate addition (Figure S5).

### Kinetic studies in whole cells

**Figure 5:**
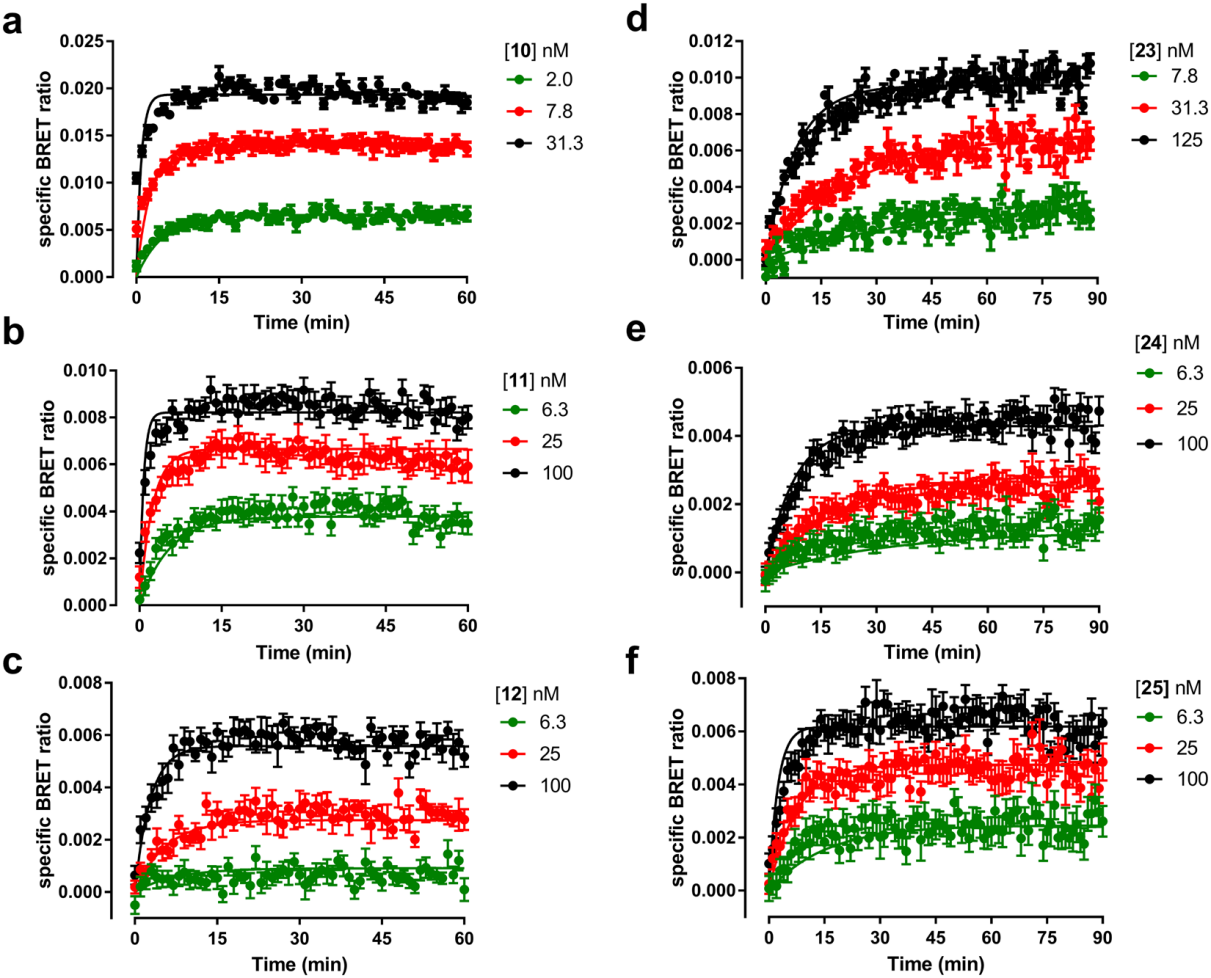
NanoBRET association kinetic studies with mepyramine and VUF13816 based fluorescent ligands. Nluc-H_1_R HEK293 cells were treated with the indicated concentrations of **10** (**a**), **11** (**b**), **12** (**c**), **23** (**d**), **24** (**e**) and **25** (**f**) and BRET monitored at room temperature every min for 60 min (**a**–**c**) or 90 min (**d**–**f**). The data shown are representative examples from four (**10**, **23**, **24**, **25**), five (**11**) or six (**12**) independent experiments performed in triplicate.

**Figure 6:**
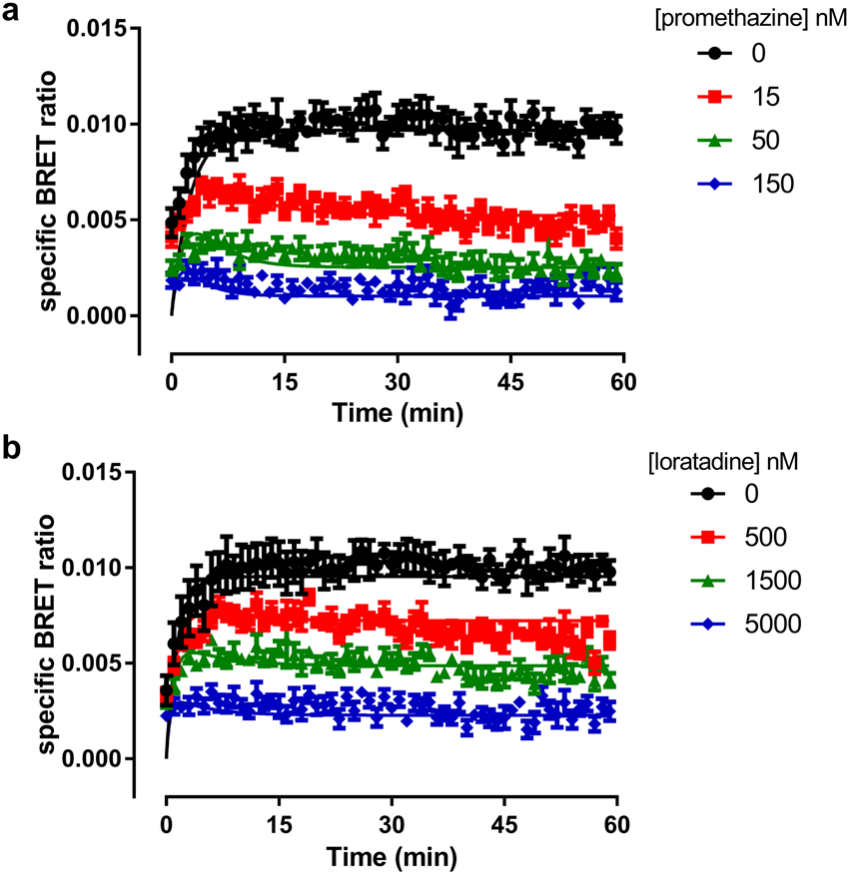
Use of fluorescent ligand **10** to determine the kinetics of unlabelled ligands. Nluc-H_1_R HEK293 cells were treated with 10 nM **10** and the indicated concentrations of promethazine (**a**) and loratidine (**b**) and BRET monitored at room temperature every min for 60 min. The data shown are representative examples from five independent experiments performed in triplicate.

The kinetics of the six fluorescent ligands was therefore evaluated in Nluc-H_1_R HEK293T cells. The specific binding of these compounds at each time point was calculated by subtracting non-specific binding from the equivalent time-matched total signal (Fig. 5). The values for the non-specific signal were obtained in the presence of a high concentration (10 μM) of doxepin as a competitor. The kinetic K_D_, k_on_ and k_off_ values obtained from the BRET data, as well as the calculated residence time for the six fluorescent H_1_R antagonists in intact cells, are shown in Table 1. These kinetic studies showed that the residence time of the fluorescent ligands based on mepyramine were approximately four fold less than those of the VUF13816 based fluorescent ligands. In addition, we observed that the kinetic K_D_ values were comparable with those obtained from the saturation binding experiments in the majority of the cases with the exception of **11**, where the kinetic K_D_ was tenfold lower than that obtained in the saturation studies. The direct measurement of kinetic parameters at a GPCR in an intact cell environment is limited to compounds that are either fluorescently or radiolabelled. To overcome this, the theoretical framework developed by Motulsky and Mahan^42^ allow the kinetic parameters of unlabelled ligands to be quantified if the kinetics of the labelled ligand are known. To demonstrate the usefulness of these novel ligands in kinetic screening of unlabelled compounds in the NanoBRET assay, we used **10** to determine the kinetic parameters of two unlabelled antagonists, promethazine and loratadine (Fig. 6 and Table 4). Kinetic data obtained in the presence of a range of competitor concentrations allowed their k_on_ and k_off_ to be determined (Table 4).

**Table 4:** Kinetic parameters of promethazine and loratadine at Nluc-H_1_R determined using NanoBRET.

	k_on_ (×10^5^ M^−1^ min^−1^)	k_off_ (min^−1^)	pK_D_
Promethazine	337.6 ± 147.1	0.18 ± 0.05	8.18 ± 0.09
Loratadine	2.2 ± 0.4	0.35 ± 0.13	5.83 ± 0.20

Kinetic parameters were obtained by monitoring the binding of 10 nM **10** in the presence of increasing concentrations of unlabelled ligand and calculated as detailed in Methods. All values represent mean ± SEM from 5 experiments performed in triplicate.

### BRET studies performed in cell membranes

One of the main advantages of the NanoBRET technique is its ability to determine ligand binding parameters in living cells. To compare the effects of an intact cellular environment on the affinity and kinetics properties of our fluorescent ligands, we also performed BRET studies in membrane preparations from Nluc-H_1_ HEK293T cells_._ In initial optimisation studies, very low levels of specific binding were observed when measuring the kinetics of conjugate **10**. To overcome this, we performed experiments in the presence of the detergent saponin (1 mg/ml), in order to disrupt membrane vesicles, which greatly improved the specific BRET ratios obtained (Figure S6). Since saponin was found to have no effect no competition binding curves and on the affinity of doxepin (Figure S6), all further studies in membranes were performed in the presence of saponin.

**Figure 7:**
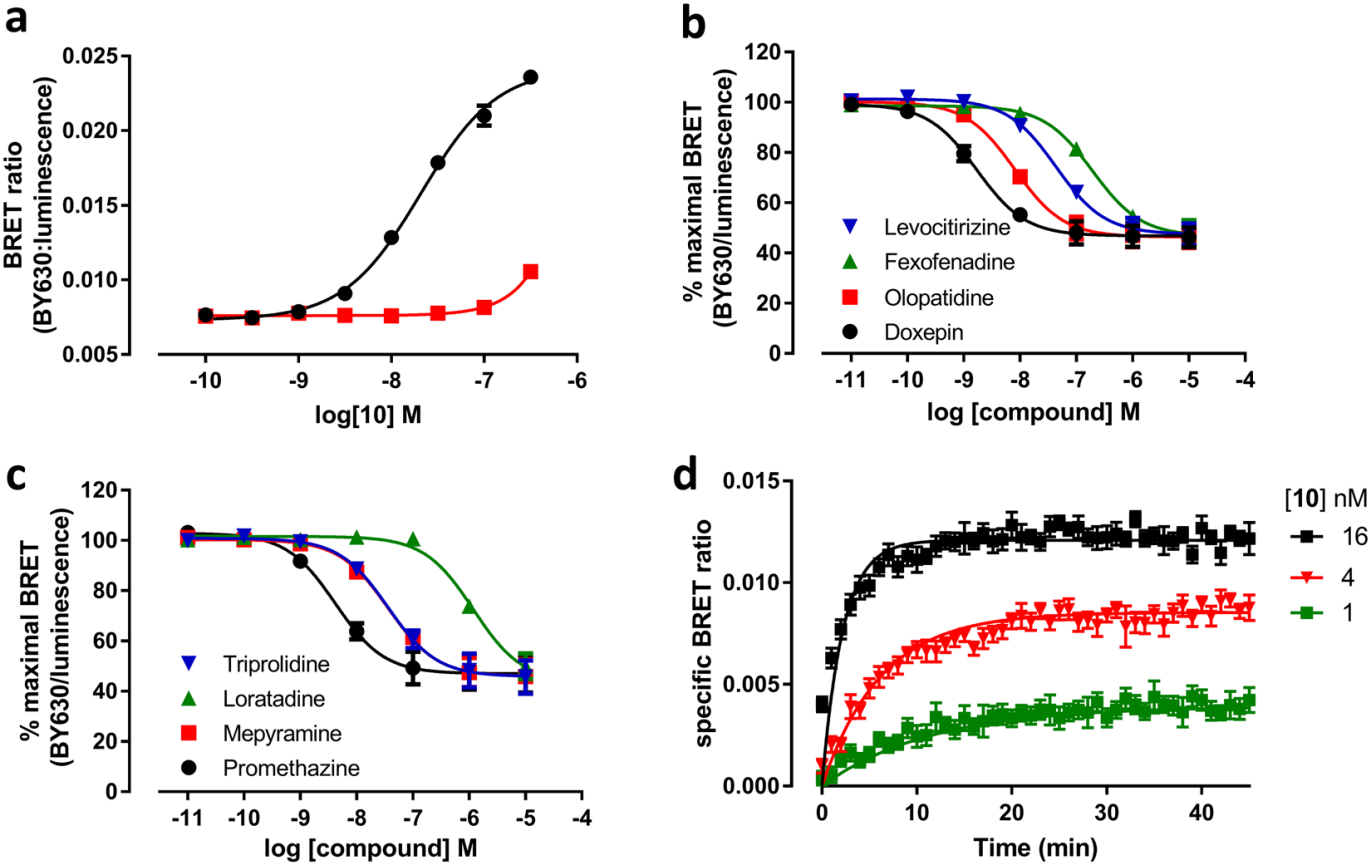
NanoBRET saturation, competition and kinetic binding studies with fluorescent ligand **10** in cell membranes. (**a**). Saturation BRET binding curves from membranes of Nluc-H_1_R HEK293 cells treated with increasing concentrations of **10** in absence (black circles) or presence (red squares) of 10 µM mepyramine. The data shown are representative of five independent experiments performed in triplicate. (**b** and **c**) Inhibition of BRET signal in membranes of Nluc-H_1_R HEK293T cells treated with 25 nM **10** and increasing concentrations of unlabelled ligands; tripolidine, loratadine, mepyramine and promethazine (**b**) or levocetirizine, fexofenadine, olopatadine and doxepin (**c**). Data were normalized to the maximal BRET ratio in each experiment. Data points are combined mean ± SEM from four separate experiments performed in triplicate (**d**). Membranes of HEK293T cells stably expressing Nluc-H_1_R treated with various concentrations of **10** were monitored by BRET at room temperature every min for 30 min. All experiments using membranes expressing Nluc-H_1_R were performed in the presence of 1 mg/ml saponin. The data shown are representative of four independent experiments performed in triplicate.

Saturation binding curves for **10** obtained in cell membranes from Nluc-H_1_R HEK293T cells obtained in the presence and absence of 10 μM of unlabelled mepyramine are shown in Fig. 7. As in cells, compound **10** showed a clear concentration-dependent and saturable increase in BRET signal, with a pK_D_ value of 7.54 ± 0.04 (n = 5). We also used **10** to perform competition binding assays in cell membranes with the same eight H_1_R antagonists as used previously (Fig. 7). The competition-binding pK_i_ values obtained (Table 3) for unlabelled antagonists were comparable with those obtained on intact cells (R^2^ = 0.96) although some minor differences were seen for individual compounds, with a trend for compounds to show higher affinity in membranes compared to whole cells. The affinity of promethazine, doxepin, olopatadine and levocetirizine was significantly higher (p < 0.05, unpaired t-test) in membranes than whole cells, whilst that for loratadine was lower (p < 0.05, unpaired t-test). Finally, we obtained the kinetic constants of **10** in membranes from cells expressing Nluc-H_1_ (Fig. 7). The k_on_ (19.64 ± 4.12 × 10^6^ M^−1^.min^−1^) and the k_off_ (0.065 min^−1^) determined in membranes were both signifcantly slower (p < 0.05, unpaired t-test) than those obtained in intact cells, but there was no difference in the calculated affinity (pK_D_ = 8.45 ± 0.12, n = 4, p > 0.05, unpaired t-test).

## Discussion

The recent development of fluorescent ligands has enabled many aspects of receptor pharmacology and function to be studied^14,43^. Here we have increased the range and improved the properties of fluorescent ligands available to study the H_1_R by synthesising six new ligands based on two different orthosteric binding moieties (mepyramine and VUF13816). We have created a series of new, high affinity fluorescent H_1_R ligands suitable for use in both confocal imaging and, in particular, in NanoBRET assays for monitoring ligand binding in living cells.

To date, there have been two reports on fluorescent ligands for the H_1_R based on the prototypic antagonist mepyramine^25,26^, but in the case of mepyramine-X-BODIPY630/650 its lipophilic nature meant it was unsuitable for confocal microscopy^26^. Using a previous strategy to develop new fluorescent ligands for the adenosine A_3_ receptor^28,44^, here we replaced the six carbon linker between the mepyramine and the BODIPY630/650 fluorophore^26^ with di- or tripeptides. Introduction of these peptide linkers resulted in some loss in affinity compared to the previously described fluorescent mepyramine (mepyramine-X-BODIPY630/650; pK_D_ = 8.9^26^), but more importantly improved their imaging properties. All three new mepyramine fluorescent ligands allowed clear visualisation of membrane binding by confocal microscopy that could be prevented by a high concentration of unlabelled mepyramine. This was in direct contrast to mepyramine-X-BODIPY630/650, where intracellular accumulation of the fluorescent ligand masked the detection of specific membrane binding by confocal microscopy. Intracellular accumulation of fluorescent ligands has been previously observed as problematic with ligands for the β_1_ adrenoceptor^17^, cannabinoid CB_1_ receptor^20^ and µ opioid receptor^37^.

We also synthesised a second series of fluorescent ligands based on a small molecule (VUF13816, MW = 267) that was first identified in a virtual screen of the H_1_R crystal structure^34^. Incorporation of peptide linkers and the BODIPY630/650-X fluorophore onto this molecule resulted in three fluorescent ligands that retained similar affinity at the H_1_R to the parent compound (pK_i_ = 8.20^34^). VUF13816 is structurally different from any other H_1_R ligand, although molecular modelling indicated that it makes similar contacts in the receptor to doxepin^34^. There was little difference in the affinity of the three VUF13816-based fluorescent ligands, even though they contained more diverse peptide linkers than the mepyramine-based ligands. As with the mepyramine based derivatives, the VUF13816-based compounds were also suitable for confocal imaging. The success of these ligands, in particular those based on VUF13816, validates the combined virtual screening and ligand design as a more structured route to fluorescent ligand design.

Both the mepyramine and VUF13816 based fluorescent ligands used in the study had similar affinity values to the parent compounds. However, for small molecule ligands for Class A GPCRs, addition of linker and fluorophore significantly increases the size and molecular complexity of the ligand. These compounds should therefore be treated as new pharmacological entities^27^, as often either increases^28,36^ or decreases^45,46^ in affinity are seen. Studies examining the influence of both the linker and fluorophore on the affinity of a fluorescent ligand have demonstrated that they can influence affinity in multiple different ways, with both the linker and flurophore playing a role in determining the final affinity of a compound^27,28,36,38,47^.

To further extend the use of the new fluorescent ligands to examine receptor binding kinetics, we examined their utility in a recently described NanoBRET proximity assay^40^. This assay utilises the extremely bright luciferase NanoLuc^48^ that is fused to the N-terminus of H_1_R. The distance constraints of energy transfer^49,50^ mean that BRET can only occur when the fluorescent ligand is in very close proximity to the receptor (i.e. <10 nm). Association kinetic binding assays using [^3^H]mepyramine indicated that the N/uc-H_1_R showed similar binding kinetics and affinity for mepyramine, suggesting that the N terminal tag did not significantly affect the receptor binding properties. The affinity of all six fluorescent ligands was measured using NanoBRET saturation assays, and the values obtained were consistent with those from radioligand binding and calcium assays. The affinities of eight unlabelled antagonists were also determined in competition binding assays using fluorescent ligands **10** and **23** as tracers. Two compounds, olopatadine and mepyramine, showed small but significant differences in their affinity depending on the fluorescent ligand used. Similar probe dependence has been observed previously for the adenosine A_3_ receptor^40^ and may reflect potential allosteric interactions across a dimeric interface^24^; the H_1_R has been previously shown to form homodimers^51^.

The NanoBRET binding assay has the advantage that it is performed in intact cells that preserve the potential for allosteric interactions with intracellular signalling proteins^10–12^. In order to investigate the influence of an intact cellular environment on the affinity of unlabelled antagonists, NanoBRET assays were also performed in cell membranes. There was a general trend for the affinities obtained in membranes to be slightly higher than those deduced in intact cells. This may be due to the presence of phosphate in the buffer used for the membrane binding assays which has been shown to be a positive modulator of ligand binding at the H_1_R^32^.

It is becoming increasingly clear that determining binding kinetics is important in the development of new therapeutics^9,52,53^, and certain kinetic profiles are desirable depending on the disease being targeted^54,55^. The determination of binding kinetics at a GPCR is often achieved through radioligand binding assays, where every time point requires a separate binding reaction that needs to be terminated before measurements can be made. This is time consuming and has led to the development of assays that allow continuous monitoring of binding, such as assays using a scintillation proximity assay for radioligands^56,57^. Greater resolution can be obtained, however, with proximity techniques such as the BRET assay used here or using fluorescence resonance energy transfer^23,25^. In the present study, the kinetic parameters of six fluorescent antagonists were determined. In equilibrium NanoBRET binding assays all of the fluorescent ligands showed similar affinities. However, the use of kinetics revealed that the VUF13816 based compounds had a slower off rate than their mepyramine counterparts. As a consequence, the VUF13816 based compounds had a residence time at the receptor that was *circa* four times longer than the mepyramine based compounds. The binding kinetics of compound **10** were also determined in membrane preparations where there were differences in both the association (19.64 × 10^6^ M^−1^.min^−1^ and 46.4 × 10^6^ M^−1^.min^−1^; cells and membranes, respectively) and dissociation rate constants (0.065 min^−1^ and 0.214 min^−1^; cells and membranes, respectively). Measuring the kinetics of a labelled compounds limits this to a small set of ligands for a given receptor, to overcome this the method developed by Motulsky and Mahan can be employed to determine the kinetics of unlabelled compounds. This was successfully achieved using the NanoBRET assay in combination with compound **10** for two unlabelled H_1_R antagonists, promethazine and loratadine. It was found that both unlabelled ligand displayed similar k_off_ rates but different k_on_ rates which is reflected in their affinity at equilibrium. The NanoBRET assay has also recently been used to resolve the kinetics of unlabelled ligands at the free fatty acid receptor 2 in cell membranes^58^. The ability to also perform these measurements in whole cells opens up the potential to assess the effect of cellular environments on rate constants as it has been shown that for some receptors that an intact cell environment can alter the binding kinetics^59^.

This study demonstrates the broad utility of fluorescent ligands for studying the H_1_R. The use of fluorescent ligands and the NanoBRET assay allows multiple aspects of receptor pharmacology to be studied in ways that are difficult with traditional assays. These can provide important insights in the function of GPCRs and into the characteristics of lead compounds for drug discovery.

## Methods

### Experimental Section

#### Chemistry

Chemicals and solvents of an analytical grade were purchased from commercial suppliers and used without further purification. 1,2-Diaminoethane trityl resin was purchased from Sigma Aldrich. 6-(((4,4-Difluoro-5-(2-thienyl)-4-bora-3a,4a-diaza-s-indacene-3-yl)styryloxy)acetyl)aminohexanoic acid, succinimidyl ester (BODIPY 630/650-X-SE) was purchased from Molecular Probes® (Invitrogen, UK). Unless otherwise stated, reactions were carried out at ambient temperature. Reactions were monitored by thin layer chromatography on commercially available pre-coated aluminium-backed plates (Merck Kieselgel 60 F254). Visualization was by examination under UV light (254 and 366 nm), or staining with KMnO_4_ dip. Flash chromatography was performed using Merck Kieselgel 60, 230–400 mesh (Merck KgaA, Darmstadt, Germany) on a Biotage Flashmaster II system. ^1^H NMR spectra were recorded on a Bruker-AV 400 at 400.13 MHz or a Bruker AV(II) 500 at 503.13 MHz. ^13^C NMR spectra were recorded on a Bruker AV(II) 500 with a dual (CH) cryoprobe at 125.8 MHz. Solvents used for NMR analysis (reference peaks listed) were DMSO-*d*_6_ ((CHD_2_)_2_SO at δ_H_ 2.50 ppm, (CD_3_)_2_SO at 39.52 ppm) and CDCl_3_ (CHCl_3_ at δ_H_ 7.26 ppm, CDCl_3_ at 77.16). Chemical shifts (δ) are recorded in parts per million (ppm). Coupling constants (*J)* are recorded in Hz and the significant multiplicities described by singlet (s), doublet (d), triplet (t), quadruplet (q), broad (br), and multiplet (m). Spectra were assigned using appropriate COSY, DEPT, HSQC and HMBC sequences. High resolution mass spectra (HRMS) – time of flight, electrospray (TOF ES +/−) were recorded on a Waters 2795 separation module/micromass LCT platform. RP-HPLC was performed using a Waters 2767 sample manager, Waters 2525 binary gradient module, and visualized at 254 nm and 366 nm with a Waters 2487 dual wavelength absorbance detector. Spectra were analyzed using MassLynx. Analytical reversed-phase high-performance liquid chromatography (RP-HPLC) was used to ensure that the purity of compounds tested in biological systems was ≥ 95% (the retention times (*R*_t_) of these compounds are reported). Analytical RP-HPLC was performed using a YMC-Pack C8 column (150 mm × 4.6 mm × 5 μm) at a flow rate of 1 mL/min, and using a method of 0–2 min 10% solvent B in solvent A, 2–25 min gradient of 10% to 90% solvent B in solvent A, 25–27 min held at 90% B in solvent A, 27–29 min gradient of 90% to 10% solvent B in solvent A, 29–33 min held at 10% solvent B in solvent A (solvent A = 0.05% TFA in H_2_O, solvent B = 0.05% TFA in 9:1 *v*:*v* CH_3_CN:H_2_O).

#### Methyl 3-((2-((4-methoxybenzyl)(pyridin-2-yl)amino)ethyl)(methyl)amino)propanoate (2)

Mepyramine (**1**) was purchased as the dimaleate salt. Prior to demethylation, mepyramine dimaleate salt was dissolved in dichloromethane and sequentially washed with saturated hydrogen bicarbonate and water. The dichloromethane layer was dried over magnesium sulfate, filtered and the solvent evaporated under reduced pressure to give mepyramine free base as a colourless oil. Mepyramine (free base) (22.7 g, 79.5 mmol) was dissolved in 300 mL of 1,2-dichloroethane and a small amount (approx. 20–30 mg) of sodium hydrogen bicarbonate was added. The solution was cooled to 0 °C and 1-chloroethyl chloroformate (25 g, 175 mmol) was added in 5 equal portions over 2 h. The reaction mixture was then heated at 60 °C for 3 h. The solution was cooled to rt, filtered, and the filtrate evaporated under reduced pressure. Methanol (350 mL) was added to the orange residue obtained and the resultant solution refluxed for 2 h. The solvent was removed under reduced pressure, the residue treated with 2 M aq. sodium hydroxide solution and extracted three times with ethyl acetate. The ethyl acetate portions were combined, dried over magnesium sulfate, filtered and evaporated under reduced pressure to give the crude product as an orange oil. Purification by flash silica column chromatography (0.5% to 5% 7 M ammonia in methanol / ethyl acetate) gave *N*^1^-(4-methoxybenzyl)-*N*^2^-methyl-*N*^1^-(pyridin-2-yl)ethane-1,2-diamine (1.428 g, 5.27 mmol, 7%) as a colourless oil. ^1^H NMR (400 MHz, CDCl_3_) δ 2.41 (s, 3 H, NHCH_3_), 2.80 (t, *J* = 6.6 Hz, 2 H, CH_2_CH_2_), 3.67 (t, *J* = 6.6 Hz, 2 H, CH_2_CH_2_), 3.76 (s, 3 H, OCH_3_), 4.70 (s, 2 H, CH_2_Ar), 6.48 (d, *J* = 8.6 Hz, 1 H, Ar pyridyl), 6.52 (m, 1 H, Ar pyridyl), 6.82 (d, *J* = 8.7 Hz, 2 H, Ar benzyl), 7.14 (d, *J* = 8.7 Hz, 2 H, Ar benzyl), 7.37 (m, 1 H, Ar pyridyl), 8.15 (m, 1 H, Ar pyridyl). ^13^C NMR (100 MHz, CDCl_3_) δ 36.65, 48.28, 49.92, 51.51, 55.32, 106.03, 111.95, 114.04, 128.10, 130.69, 137.29, 148.04, 158.53, 158.70. HRMS calculated for C_16_H_22_N_3_O 272.1757 (M + H)^+^, found 272.1740. To a stirred solution of *N*^1^-(4-methoxybenzyl)-*N*^2^-methyl-*N*^1^-(pyridin-2-yl)ethane-1,2-diamine (1.428 g, 5.27 mmol) in 1,2-dichloroethane (8 mL) was added methyl acrylate (2.2 mL, approx. 26 mmol, approx. 5 equiv), and the mixture stirred at 80 °C for 3 h. The solvent was removed under reduced pressure to give an orange oil. Purification by flash silica column chromatography (0.1% to 0.5% 7 M ammonia in methanol / ethyl acetate) gave **2** (1.602 g, 4.49 mmol, 85%) as a pale yellow oil. ^1^H NMR (400 MHz, CDCl_3_) δ 2.27 (s, 3 H, NCH_3_), 2.45 (t, *J* = 7.2 Hz, 2 H, CH_2_CH_2_), 2.57 (t, *J* = 7.3 Hz, 2 H, CH_2_CH_2_), 2.73 (t, *J* = 7.2 Hz, 2 H, CH_2_CH_2_), 3.62 (t, *J* = 7.3 Hz, 2 H, CH_2_CH_2_), 3.65 (s, 3 H, CO_2_CH_3_), 3.78 (s, 3 H, OCH_3_), 4.69 (s, 2 H, CH_2_Ar), 6.44 (d, *J* = 8.8 Hz, 1 H, Ar pyridyl), 6.52 (m, 1 H, Ar pyridyl), 6.84 (d, *J* = 8.6 Hz, 2 H, Ar benzyl), 7.16 (d, *J* = 8.6 Hz, 2 H, Ar benzyl), 7.37 (m, 1 H, Ar pyridyl), 8.16 (m, 1 H, Ar pyridyl). ^13^C NMR (100 MHz, CDCl_3_) δ 32.61, 42.49, 46.49, 51.51, 51.64, 53.27, 54.66, 55.33, 105.83, 111.76, 114.01, 128.23, 130.89, 137.24, 148.12, 158.23, 158.71, 173.09. HRMS calculated for C_20_H_28_N_3_O_3_ 358.2125 (M + H)^+^, found 358.2139.

#### 3-((2-((4-Methoxybenzyl)(pyridin-2-yl)amino)ethyl)(methyl)amino)propanoic acid (3)

To a stirred solution of **2** (1.602 g, 4.49 mmol) in THF at 0 °C was added dropwise a solution of 0.2 M aq. lithium hydroxide (8.98 mmol, 45 mL). The solution was stirred at 0 °C, monitored by LCMS, and after 2 h the reaction was complete. The solution was adjusted to pH 7 using 1 M aq. HCl, gently evaporated under reduced pressure to constant volume (to remove the THF), and then freeze-dried to afford a white solid. Chloroform (approx. 100 mL) was added, the mixture sonicated, and then filtered. The filtrate was evaporated to dryness under reduced pressure, to yield approximately 2 g of white solid. To further desalt the sample, the solid was re-dissolved in chloroform and washed with water, the water layer extracted three times with chloroform, the chloroform extracts combined, dried, and evaporated to dryness to give **3** as a white solid (1.696 g). Assuming one equivalent of lithium chloride, this gave 4.4 mmol, 90% yield. ^1^H NMR (400 MHz, CDCl_3_) δ 2.45 (t, *J* = 6.1 Hz, 2 H, CH_2_CH_2_), 2.53 (s, 3 H, NCH_3_), 2.89 (m, 4 H, CH_2_CH_2_, CH_2_CH_2_), 3.76 (s, 3 H, OCH_3_), 3.86 (t, *J* = 6.6 Hz, 2 H, CH_2_CH_2_), 4.60 (s, 2 H, CH_2_Ar), 6.48 (d, *J* = 8.5 Hz, 1 H, Ar pyridyl), 6.56 (m, 1 H, Ar pyridyl), 6.83 (d, *J* = 8.4 Hz, 2 H, Ar benzyl), 7.11 (d, *J* = 8.7 Hz, 2 H, Ar benzyl), 7.37 (m, 1 H, Ar pyridyl), 8.13 (m, 1 H, Ar pyridyl), 10.36 (br s, 1 H, CO_2_H). ^13^C NMR (100 MHz, CDCl_3_) δ 30.43, 40.70, 44.89, 52.00, 53.38, 53.65, 55.36, 106.42, 112.62, 114.24, 128.05, 129.82, 137.58, 147.98, 157.89, 158.93, 174.20. HRMS calculated for C_19_H_26_N_3_O_3_ 344.1969 (M + H)^+^, found 344.1951.

#### General Procedure A (Supplementary Figure 1a): Solid-phase synthesis of 7, 8, 9, 20, 21 and 22

1,2-Diaminoethane trityl resin (substitution 1.2–1.7 mmol/g depending on batch) (200–350 mg, assume 0.34–0.60 mmol) was swelled in DMF for 4 h, drained, then a solution of the first Fmoc-amino acid (3 equiv), HBTU (3 equiv) and DIPEA (6 equiv) in DMF (approx. 3 mL) was added and left for 1–2 h. The resin was drained, washed with DMF, and double coupled using the same Fmoc-amino acid/HBTU/DIPEA coupling procedure. The resin was drained, washed with DMF, and a solution of acetic anhydride (500 µL) and DIPEA (500 µL) in DMF was added to cap any unreacted sites. The resin loading was estimated by determining the Fmoc-content of the resin and calculated to be 0.5–0.7 mmol/g across different resin batches and experiments. The resin was then treated with three repeated cycles of 20% *v/v* piperidine in DMF for 10 min, washed with DMF, then the second Fmoc-amino acid (3 equiv rel. to resin loading) was coupled using HBTU (3 equiv) and DIPEA (6 equiv) in DMF (approx. 3 mL). The Fmoc groups was again cleaved using 20% *v/v* piperidine/DMF as described above, and then any additional amino acids (or Fmoc-4-aminobutyric acid) were added as required using the same HBTU coupling/Fmoc cleavage standard solid-phase peptide synthesis procedure described above. After the last required amino acid was coupled and Fmoc deprotected, either the mepyramine derivative **3** or VUF13816 derivative **16** (1.1 equiv rel to resin loading) was coupled to the resin-bound peptide using HATU (1.1 equiv) and DIPEA (4 equiv) in DMF. The resin was washed with DMF then DCM, dried under nitrogen and vacuum, and the product cleaved from the resin using 5% TFA/DCM. The filtrate solvent was evaporated, and if the peptide contained *t*-butyl side chain protecting group(s), this residue was dissolved in 1:1 *v/v* DCM/TFA and stirred for 2–4 hours to allow for amino acid side-chain deprotection.

#### (*S*)-*N*-(2-aminoethyl)-2-((*S*)-2-(3-((2-((4-methoxybenzyl)(pyridin-2-yl)amino)ethyl)(methyl)amino)propanamido)propanamido)propanamide (7)

1,2-Diaminoethane trityl resin (560 mg) was reacted according to General Procedure A, and the crude resin-cleaved residue purified using preparative RP-HPLC to give **7** (166 mg) as a white solid. ^1^H NMR (400 MHz, DMSO-*d*_6_) δ 1.20 (m, 6 H, ala CH_3_), 2.67 (t, *J* = 7.4 Hz, 2 H, COCH_2_), 2.83–2.87 (m, 5 H, NCH_3_, CH_2_NH_2_), 3.27–3.32 (m, 4 H, NHCH_2_, MeNCH_2_), 3.39 (m, 2 H, MeNCH_2_), 3.72 (s, 3 H, OMe), 3.90 (t, J = 6.5 Hz, 2 H, ArNCH_2_), 4.19 (m, 1 H, ala CH), 4.30 (m, 1 H, ala CH), 4.65 (s, 2 H, ArCH_2_), 6.66–6.73 (m, 2 H, ArH), 6.90 (d, *J* = 8.7 Hz, 2 H, ArH PMB), 7.15 (d, *J* = 8.7 Hz, 2 H, ArH PMB), 7.54 (m, 1 H, ArH), 7.92 (br s, 2 H, NH_2_), 8.08 (t, *J* = 5.5 Hz, 1 H, NHCH_2_), 8.12–8.15 (m, 2 H, ala NH, ArH), 8.40 (d, *J* = 7.8 Hz, 1 H, ala NH). ^13^C NMR (125 MHz, DMSO-*d*_6_) δ 17.98, 18.09, 29.35, 36.46, 38.43, 43.47, 48.28, 48.38, 50.91, 51.87, 53.36, 55.08, 107.22, 112.68, 114.08, 127.99, 129.54, 138.40, 146.77, 157.10, 158.44, 168.90, 171.89, 172.76. HRMS calculated for C_27_H_42_N_7_O_4_ 528.3293 (M + H)^+^, found 528.3271.

#### (*S*)-*N*-(2-aminoethyl)-3-hydroxy-2-((10 *S*,13 *S*)-1-(4-methoxyphenyl)-5,10,13-trimethyl-8,11-dioxo-2-(pyridin-2-yl)-2,5,9,12-tetraazatetradecan-14-amido)propanamide (8)

1,2-Diaminoethane trityl resin (222 mg) was reacted according to the General Procedure A to give the resin-cleaved crude **8** (120 mg) as a yellow oil. Analytical RP-HPLC and MS analysis showed approximately 50% of the sample to be the desired product and 50% the product but with the *p*-methoxybenzyl group cleaved. Therefore, a small amount of the crude **8** (10 mg) was purified by preparative RP-HPLC to give a pure sample (4.4 mg, 7.2 µumol) free from any *p*-methoxybenzyl cleaved side-product. HRMS calculated for C_30_H_47_N_8_O_6_ 615.3613 (M + H)^+^, found 615.3643. This purified material was used to couple to BODIPY 630/650-X-SE.

#### *N*-((*S*)-1-(((*S*)-1-((2-aminoethyl)amino)-1-oxopropan-2-yl)amino)-1-oxopropan-2-yl)-4-(3-((2-((4-methoxybenzyl)(pyridin-2-yl)amino)ethyl)(methyl)amino)propanamido)butanamide (9)

1,2-Dichloroethane trityl resin (180 mg) was reacted according to the General Procedure A to give the resin-cleaved crude **9** (105 mg) as a white solid. The crude residue was purified using preparative HPLC to give **9** (65 mg) as a white solid. ^1^H NMR (400 MHz, DMSO-*d*_6_) δ 1.20 (m, 6 H, ala CH_3_), 1.62 (m, 2 H, CH_2_), 2.13 (t, *J* = 7.4 Hz, 2 H, COCH_2_), 2.60 (t, *J* = 7.1 Hz, 2 H, COCH_2_), 2.83–2.87 (m, 5 H, NCH_3_, CH_2_NH_2_), 3.04 (m, 2 H, NHCH_2_), 3.27–3.33 (m, 4 H, NHCH_2_, MeNCH_2_), 3.39 (m, 2 H, MeNCH_2_), 3.72 (s, 3 H, OMe), 3.90 (t, *J* = 6.8 Hz, 2 H, ArNCH_2_), 4.14–4.26 (m, 2 H, ala CH), 4.65 (s, 2 H, ArCH_2_), 6.66–6.71 (m, 2 H, ArH), 6.89 (d, *J* = 8.7 Hz, 2 H, ArH PMB), 7.15 (d, *J* = 8.7 Hz, 2 H, ArH PMB), 7.53 (m, 1 H, ArH), 7.87 (br s, 2 H, NH_2_), 8.00–8.08 (m, 3 H, ala NH, ala NH, NHCH_2_), 8.12 (m, 1 H, ArH), 8.19 (t, *J* = 5.4 Hz, 1 H, NHCH_2_). ^13^C NMR (125 MHz, DMSO-*d*_6_) δ 17.84, 17.85, 25.19, 29.30, 32.47, 36.43, 38.22, 38.44, 43.44, 48.35, 50.90, 52.06, 53.45, 55.07, 107.11, 112.67, 114.07, 127.96, 129.57, 138.26, 146.95, 157.21, 158.40, 168.96, 171.97, 172.27, 171.78. HRMS calculated for C_31_H_49_N_8_O_5_ 613.3820 (M + H)^+^, found 613.3790.

#### General Procedure B: Synthesis of 10, 11, 12, 23, 24, 25 (Supplementary Figure 1b)

The amine congener (as the TFA salt) was dissolved in methanol and Amberlyst 21 resin was added, the solution filtered and the filtrate evaporated to give the neutralized free amine congener. This free amine (1–5 equiv) was dissolved in DMF and BODIPY 630/650-X-SE (1 equiv) was added and the reaction stirred at rt for 2 h in the dark. The solvent was removed under reduced pressure and the residue purified by semi-preparative RP-HPLC.

#### 6-(2-(4-((*E*)-2-(5,5-difluoro-7-(thiophen-2-yl)-5*H*-4λ^4^,5λ^4^-dipyrrolo[1,2-*c*:2′,1′-*f*][1,3,2]diazaborinin-3-yl)vinyl)phenoxy)acetamido)-*N*-((10 *S*,13 *S*)-1-(4-methoxyphenyl)-5,10,13-trimethyl-8,11,14-trioxo-2-(pyridin-2-yl)-2,5,9,12,15-pentaazaheptadecan-17-yl)hexanamide (10)

According to General Procedure B, the neutralized amine of **7** (3.2 mg, 6 µmol) was reacted with BODIPY 630/650-X-SE (4 mg, 6 µmol) to give a blue solid. This was dissolved in methanol, filtered through a small column containing Amberlyst 21 resin, and the methanol evaporated to give **10** (3.6 mg, 3.4 µmol, 57% yield) as a blue solid. HRMS calculated for C_56_H_68_BF_2_N_10_O_7_S 1073.5049 (M + H)^+^, found 1073.5050. Analytical RP-HPLC *R*_t_ = 18.8 min.

#### 6-(2-(4-((*E*)-2-(5,5-difluoro-7-(thiophen-2-yl)-5*H*-4λ^4^,5λ^4^-dipyrrolo[1,2-*c*:2′,1′-*f*][1,3,2]diazaborinin-3-yl)vinyl)phenoxy)acetamido)-*N*-((10 *S*,13 *S*,16 *S*)-16-(hydroxymethyl)-1-(4-methoxyphenyl)-5,10,13-trimethyl-8,11,14,17-tetraoxo-2-(pyridin-2-yl)-2,5,9,12,15,18-hexaazaicosan-20-yl)hexanamide (11)

According to General Procedure B, the neutralized amine of **8** (2.2 mg, 3.6µmol) was reacted with BODIPY 630/650-X-SE (1 mg, 1.5 µmol) to give a blue solid. This was dissolved in methanol, filtered through a small column containing Amberlyst 21 resin, and the methanol evaporated to give **11** (1.0 mg, 0.86 µmol, 57% yield) as a blue solid. HRMS calculated for C_59_H_73_BF_2_N_11_O_9_S 1160.5369 (M + H)^+^, found 1160.5326. Analytical RP-HPLC *R*_t_ = 17.2 min.

#### 5,5-difluoro-3-((*E*)-4-(((15 *R*,18 *R*)-1-(4-methoxyphenyl)-5,15,18-trimethyl-8,13,16,19,24,31-hexaoxo-2-(pyridin-2-yl)-2,5,9,14,17,20,23,30-octaazadotriacontan-32-yl)oxy)styryl)-7-(thiophen-2-yl)-5*H*-4λ^4^-dipyrrolo[1,2-*c*:2′,1′-*f*][1,3,2]diazaborinin-5-uide (12)

According to General Procedure B, the neutralized amine of **9** (5 mg, 8.2 µmol) was reacted with BODIPY 630/650-X-SE (1 mg, 1.5 µmol) to give a blue solid. This was dissolved in methanol, filtered through a small column containing Amberlyst 21 resin, and the methanol evaporated to give **12** (0.7 mg, 0.6 µmol, 40% yield) as a blue solid. HRMS calculated for C_60_H_75_BF_2_N_11_O_8_S 1158.5576 (M + H)^+^, found 1158.5603. Analytical RP-HPLC *R*_t_ = 18.6 min.

#### *tert*-Butyl 4-(2-benzylphenoxy)piperidine-1-carboxylate (14)

Potassium hydroxide (403 mg, 7.2 mmol), 2-benzylphenol (445 mg, 2.4 mmol) and *tert*-butyl 4-bromopiperidine-1-carboxylate (3.1 g, 11.8 mmol) were dissolved in methanol (5 mL) and refluxed for 2 days. More potassium hydroxide (201 mg, 3.6 mmol) and *tert*-butyl 4-bromopiperidine-1-carboxylate (1.5 g, 5.7 mmol) were added and the reaction refluxed for a further day. The solvent was evaporated under reduced pressure and the residue dissolved in ethyl acetate. This was washed three times with 1 M NaOH aq. solution, once with saturated aq. brine, the ethyl acetate layer dried over MgSO4, filtered, and evaporated to give the crude product. This was purified using flash silica column chromatography (0 to 16% ethyl acetate in petroleum ether) to give **14** (246 mg, 0.67 mmol, 28% yield) as a colourless oil. ^1^H NMR (400 MHz, CDCl_3_) δ 1.15 (s, 9 H, C(CH_3_)_3_), 1.74 (m, 2 H, CH_2_), 1.86 (m, 2 H, CH_2_), 3.41 (m, 2 H, CH_2_), 3.50 (m, 2 H, CH_2_), 4.02 (s, 2 H, ArCH_2_), 4.52 (m, 1 H, CH), 6.87–6.94 (m, 2 H, ArH), 7.17–7.30 (m, 7 H, ArH). ^13^C NMR (100 MHz, CDCl_3_) δ 28.55, 30.47, 36.57, 40.45, 71.41, 79.62, 112.55, 120.62, 125.90, 127.48, 128.30, 128.88, 130.61, 131.13, 141.22, 154.94. HRMS calculated for C_23_H_30_NO_3_^+^ 368.2220 (M + H)^+^, found 368.2239.

#### Methyl 3-(4-(2-benzylphenoxy)piperidin-1-yl)propanoate (15)

14 (246 mg, 0.67 mmol) was dissolved in dichloromethane (4 mL) and trifluoroacetic acid (1 mL) was added. The mixture was stirred for 1 h at rt, then the solvent removed under reduced pressure, and the residue dissolved in ethyl acetate. The organic layer was washed three times was saturated aq. sodium bicarbonate, dried over MgSO_4_, and evaporated to give 4-(2-benzylphenoxy)piperidine (178 mg, 0.67 mmol, quantitative) as a colourless oil. This was used without purification in the next reaction. HRMS calculated for C_18_H_22_NO^+^ 268.1696 (M + H)^+^, found 268.1699.

4-(2-benzylphenoxy)piperidine (170 mg, 0.64 mmol) was dissolved in DCM and neutralized by washing with saturated aq. sodium carbonate. The DCM layer was dried and the solvent removed under reduced pressure. This residue was dissolved in 1,2-dichloroethane (1 mL) and methyl acrylate (350 µl, 3.84 mmol) was added. The reaction was heated to 60 °C and stirred for 1.5 h. The solvent was removed under reduced pressure and the residue purified by silica flash column chromatography (petroleum ether, gradient of 0 to 30% solution of [5% 7 M ammonia in methanol in 95% ethyl acetate]) to give **15** (200 mg, 0.57 mmol, 89% yield) as a pale yellow oil. ^1^H NMR (400 MHz, CDCl_3_) δ 1.83 (m, 2 H, CH_2_ pip), 1.95 (m, 2 H, CH_2_ pip), 2.36 (m, 2 H, CH_2_ pip), 2.52 (t, *J* = 7.7 Hz, 2 H, CH_2_ ethyl), 2.61 (m, 2 H, CH_2_ pip), 2.70 (t, *J* = 7.7 Hz, 2 H, CH_2_ ethyl), 3.72 (s, 3 H, OMe), 4.00 (s, 2 H, ArCH_2_), 4.39 (m, 1 H, CH), 6.85–6.91 (m, 2 H, ArH), 7.12–7.30 (m, 7 H, ArH). ^13^C NMR (100 MHz, CDCl_3_) δ 30.76, 32.36, 36.49, 50.11, 51.76, 53.66, 71.62, 112.59, 120.41, 125.84, 127.41, 128.30, 128.98, 130.60, 131.00, 141.36, 155.14, 173.14. HRMS calculated for C_22_H_28_NO_3_^+^ 354.2064 (M + H)^+^, found 354.2034.

#### 3-(4-(2-Benzylphenoxy)piperidin-1-yl)propanoic acid (16)

To a stirred solution of **15** (200 mg, 0.57 mmol) in THF (5 mL) at 0 °C was added dropwise a solution of lithium hydroxide in water (0.2 M, 1.14 mmol, 5.7 mL). The solution was stirred at 0 °C for 2 h, neutralized to pH = 7 using 1 M aq. hydrochloric acid, and the solvent removed under reduced pressure. The material was redissolved in water and freeze-dried overnight. Chloroform was added to the dried sample, and the sample filtered, the white solid washed with chloroform, the chloroform washings combined, dried over magnesium sulfate, and evaporated under reduced pressure to give **16** (180 mg, assuming one equiv. of LiCl salt, 0.47 mmol, 83%) as a pale yellow solid. ^1^H NMR (400 MHz, DMSO-*d*_6_) δ 1.60 (m, 2 H, CH_2_ pip), 1.85 (m, 2 H, CH_2_ pip), 2.28–2.34 (m, 4 H, CH_2_), 2.50 (m, 2 H, CH_2_ ethyl), 2.54 (m, 2 H, CH_2_ pip), 3.88 (s, 2 H, ArCH_2_), 4.41 (m, 1 H, CH), 6.84 (m, 1 H, ArH), 6.95 (d, *J* = 7.9 Hz, 1 H, ArH), 7.11–7.25 (m, 7 H, ArH). ^13^C NMR (100 MHz, DMSO-*d*_6_) δ 30.14, 32.51, 35.72, 49.35, 53.60, 71.11, 112.93, 120.18, 125.70, 127.48, 128.15, 128.62, 129.97, 130.60, 141.15, 154.54, 174.29. HRMS calculated for C_21_H_26_NO_3_^+^ 340.1907 (M + H)^+^, found 340.1941.

#### (*S*)-*N*-(2-aminoethyl)-2-((*S*)-2-((*S*)-2-(3-(4-(2-benzylphenoxy)piperidin-1-yl)propanamido)propanamido)propanamido)propanamide (20)

1,2-Diaminoethane trityl resin (250 mg) was reacted according to the General Procedure A, and the crude resin-cleaved residue purified using preparative RP-HPLC to give **20** (46 mg, 0.065 mmol) as a white solid. Now with a bulky group containing stereocentres attached via *N*-piperidyl substitution the compound is a mixture of conformational isomers (cis/trans across piperidyl ring) which shows some peak resolution in the ^1^H and ^13^C NMR spectra, however the analytical RP-HPLC shows one sharp peak. Where the isomer is resolved in the NMR, this is denoted with an *asterisk for the minor isomer. NMR was assigned using COSY and HSQC spectra. ^1^H NMR (400 MHz, DMSO-*d*_6_) δ 1.20–1.23 (m, 9 H, ala CH_3_), 1.74 (m, 1 H, CHH pip), 1.95 (m, 2 H, CH_2_ pip), 2.21 (m, 1 H, CHH pip), 2.58–2.70 (m, 4 H, CH_2_ pip, COCH_2_), 2.84 (m, 2 H, H_2_NCH_2_), 3.16 (m, 2 H, pipNCH_2_), 3.27–3.31 (m, 4 H, NHCH_2_, CH_2_ pip), 3.87* (s, 2 H, ArCH_2_), 3.98 (s, 2 H, ArCH_2_), 4.18 (m, 1 H, ala CH), 4.25 (m, 1 H, ala CH), 4.32 (m, 1 H, ala CH), 4.55* (m, 1 H, CH pip), 4.72 (m, 1 H, CH pip), 6.91 (m, 1 H, ArH), 6.98 (d, *J* = 8.4 Hz, 1 H, ArH), 7.05* (d, *J* = 8.6 Hz, 1 H, ArH), 7.15–7.30 (m, 7 H, ArH), 7.84 (br s, 2 H, NH_2_), 7.92 (d, *J* = 7.3 Hz, 1 H, ala NH), 8.07 (t, *J* = 5.4 Hz, 1 H, NHCH_2_), 8.17 (d, *J* = 7.3 Hz, 1 H, ala NH), 8.39 (d, *J* = 7.0 Hz, 1 H, ala NH), 9.62* (br s, 2 H, NH_2_). ^13^C NMR (125 MHz, DMSO-*d*_6_) δ 17.81, 18.05, 18.19, 26.40, 28.18, 29.40, 29.62*, 35.57*, 35.97*, 36.42, 38.42, 47.29*, 47.38, 48.16, 48.29, 49.94, 50.03*, 51.28*, 52.08, 65.40, 69.98*, 112.24, 113.16*, 120.55, 120.91*, 125.76*, 125.83, 127.54*, 127.74, 128.17*, 128.36, 128.60, 129.51, 130.13*, 130.64*, 131.16, 140.95*, 141.37, 153.87, 154.22*, 168.56, 168.63*, 171.81, 172.08, 172.69. HRMS calculated for C_32_H_47_N_6_O_5_^+^ 595.3602 (M + H)^+^, found 595.3596.

#### (*S*)-*N*^1^-(2-aminoethyl)-2-(2-(2-(3-(4-(2-benzylphenoxy)piperidin-1-yl)propanamido)acetamido)acetamido)succinamide (21)

1,2-Diaminoethane trityl resin (250 mg) was reacted according to the General Procedure A, and the crude resin-cleaved residue purified using preparative RP-HPLC to give **21** (40 mg, 0.055 mmol) as a white solid. ^1^H NMR (400 MHz, DMSO-*d*_6_) δ 1.75 (m, 1 H, CHH pip), 1.96 (m, 2 H, CH_2_ pip), 2.12 (m, 1 H, CHH pip), 2.52 (m, 2 H, asn CH_2_), 2.64–2.71 (m, 4 H, CH_2_ pip, COCH_2_), 2.87 (m, 2 H, H_2_NCH_2_), 3.18 (m, 2 H, CH_2_ pip), 3.27–3.37 (m, 4 H, NHCH_2_, pipNCH_2_), 3.74–3.80 (m, 4 H, gly CH_2_), 3.87* (s, 2 H, ArCH_2_), 3.98 (s, 2 H, ArCH_2_), 4.50 (m, 1 H, asn CH), 4.56* (m, 1 H, CH pip), 4.73 (m, 1 H, tyr CH), 6.91 (m, 1 H, ArH), 6.99 (m, 1 H, ArH), 7.04* (m, 1 H, ArH), 7.16–7.28 (m, 7 H, ArH), 7.48 (s, 2 H, asn NH_2_), 7.83 (br s, 2 H, CH_2_NH_2_), 8.04 (t, *J* = 6.0 Hz, 1 H, NHCH_2_), 8.16 (d, *J* = 6.8 Hz, 1 H, asn NH), 8.25 (t, *J* = 5.7 Hz, 1 H, gly NH), 8.48 (br m, 1 H, gly NH), 9.71* (br s, 2 H, NH_2_). ^13^C NMR (125 MHz, DMSO-*d*_6_) δ 26.40, 28.21, 28.49*, 29.43, 29.65*, 35.61, 35.97, 36.41, 37.13, 38.69, 42.00, 42.07, 47.39, 48.61*, 49.79, 50.02*, 51.29*, 52.09, 54.93*, 65.43, 70.02*, 112.27, 113.18*, 113.64*, 116.03*, 118.41*, 120.56, 120.79*, 120.90*, 125.84, 127.58*, 127.74, 128.19*, 128.37, 128.61, 129.54, 130.14*, 130.64*, 131.15, 140.96*, 141.37, 153.89, 154.22*, 168.68, 169.28, 169.34, 171.60, 171.72. HRMS calculated for C_31_H_44_N_7_O_6_^+^ 610.3348 (M + H)^+^, found 610.3373.

#### (*S*)-*N*^1^-((*S*)-1-(((2 *S*,3 *S*)-1-((2-aminoethyl)amino)-3-hydroxy-1-oxobutan-2-yl)amino)-1-oxopropan-2-yl)-2-(3-(4-(2-benzylphenoxy)piperidin-1-yl)propanamido)succinamide (22)

1,2-Diaminoethane trityl resin (250 mg) was reacted according to the General Procedure A, and the crude resin-cleaved residue purified using preparative RP-HPLC to give **22** (55 mg, 0.07 mmol) as a white solid. HRMS calculated for C_34_H_50_N_7_O_7_^+^ 668.3766 (M + H)^+^, found 668.3737.

#### *N*-((5 *S*,8 *S*,11 *S*)-15-(4-(2-benzylphenoxy)piperidin-1-yl)-5,8,11-trimethyl-4,7,10,13-tetraoxo-3,6,9,12-tetraazapentadecyl)-6-(2-(4-((*E*)-2-(5,5-difluoro-7-(thiophen-2-yl)-5*H*-4λ^4^,5λ^4^-dipyrrolo[1,2-*c*:2′,1′-*f*][1,3,2]diazaborinin-3-yl)vinyl)phenoxy)acetamido)hexanamide (23)

According to General Procedure B, the neutralized amine of **20** (2.4 mg, 4 µmol) was reacted with BODIPY 630/650-X-SE (1.25 mg, 1.9 µmol) to give a blue solid. This was dissolved in methanol, filtered through a small column containing Amberlyst 21 resin, and the methanol evaporated to give **23** (1.4 mg, 1.2 µmol, 65%) as a blue solid. HRMS calculated for C_61_H_73_BF_2_N_9_O_8_S 1140.5358 (M + H)^+^, found 1140.5352. Analytical RP-HPLC *R*_t_ = 20.9 min.

#### (*S*,*E*)-2-(2-(2-(3-(4-(2-benzylphenoxy)piperidin-1-yl)propanamido)acetamido)acetamido)-*N*^1^-(2-(6-(2-(4-(2-(5,5-difluoro-7-(thiophen-2-yl)-5*H*-4λ^4^,5λ^4^-dipyrrolo[1,2-*c*:2′,1′-*f*][1,3,2]diazaborinin-3-yl)vinyl)phenoxy)acetamido)hexanamido)ethyl)succinamide (24)

According to General Procedure B, the neutralized amine of **21**(3 mg, 4.9 µmol) was reacted with BODIPY 630/650-X-SE (1.25 mg, 1.9 µmol) to give a blue solid. This was dissolved in methanol, filtered through a small column containing Amberlyst 21 resin, and the methanol evaporated to give **24** (1.7 mg, 1.4 µmol, 73%) as a blue solid. HRMS calculated for C_60_H_70_BF_2_N_10_O_9_S 1155.5104 (M + H)^+^, 1155.5100. Analytical RP-HPLC *R*_t_ = 20.4 min.

#### (*S*)-2-(3-(4-(2-benzylphenoxy)piperidin-1-yl)propanamido)-*N*^1^-((15 *S*,18 *S*)-1-(4-((*E*)-2-(5,5-difluoro-7-(thiophen-2-yl)-5*H*-4λ^4^,5λ^4^-dipyrrolo[1,2-*c*:2′,1’-*f*][1,3,2]diazaborinin-3-yl)vinyl)phenoxy)-15-((*S*)-1-hydroxyethyl)-2,9,14,17-tetraoxo-3,10,13,16-tetraazanonadecan-18-yl)succinamide (25)

According to General Procedure B, the neutralized amine of **22** (3 mg, 4.5 µmol) was reacted with BODIPY 630/650-X-SE (1.25 mg, 1.9 µmol) to give a blue solid. This was dissolved in methanol, filtered through a small column containing Amberlyst 21 resin, and the methanol evaporated to give **25** (0.7 mg, 0.6 µmol, 37%) as a blue solid. HRMS calculated for C_63_H_76_BF_2_N_10_O_10_S 1213.5522 (M + H)^+^, found 1213.5541. Analytical RP-HPLC *R*_t_ = 20.5 min.

### Computational modelling

#### Construction of the **23**-H_1_R model

The H_1_R crystal structure (PDB-code 3RZE)^32^ was prepared by modeling missing atoms (K442, R481) and missing residues (F168-V174 in extracellular loop 2). The fused T4-lysozyme was removed and the C-terminus of C221 and N-terminus of L405 were linked and three residues upstream and downstream were minimized. All non-amino acids were removed except for the E-isomer of Doxepin (PDB-code 5EH). The complex was inserted in a POPC layer and waters and ions were added according to a previously described protocol^60^. Subsequently the system was equilibrated and simulated for 12.5 ns using GROMACS 4.6.1^61^ using the Amber ff99SB-ILDN force field^61,62^. The final MD snapshot (with an RMSD of 1.6 Å compared to the crystal structure) was used for PLANTS^63^ docking of VUF13816 with increasing segments of the AAA linker. Finally the BY630 fluorophore was manually positioned between the protein and the membrane (near the final part of the linker) and connected to the linker. The full **23** ligand was subsequently minimized while keeping the membrane and the protein rigid.

## General Pharmacology Methods

### Materials

G418, Lipofectamine and Optimem were obtained from Life Technologies (Paisley, UK) and fetal calf serum from PAA Laboratories (Wokingham, UK). Furimazine was obtained from Promega (Southampton, UK). Fexofenadine and doxepin were from Tocris Bioscience (Bristol, UK). All other chemicals and reagents were obtained from Sigma-Aldrich (Gillingham, UK). GF/C plates, Microscint-O and [^3^H]mepyramine were from Perkin Elmer (Waltham, MA,USA). 25-kDa linear polyethylenimine for transfection was from Poly- sciences (Warrington, PA, USA).

### Generation of cell lines and cell culture

The cDNA clone for the human H_1_R was obtained from the Missouri S&T cDNA Resource Centre (www.cDNA.org) and the H_1_-YFP was as used in Rose *et al*.^26^. Nluc-H_1_ was generated by amplifying the full length sequence H_1_R (with the methionine start signal removed) and fusing it in-frame with the membrane signal sequence of 5HT_3A_ receptor and Nluc. Chinese hamster ovary (CHO; obtained from ATCC, Manassas, VA, USA) cells were transfected with cDNA encoding the H_1_R or H_1_-YFP and human embryonic kidney (HEK293T; obtained from ATCC) cells were transfected with Nluc-H_1_ using Lipofectamine (Life Technologies, Paisley, UK) according to the manufacturer’s instructions. Cells were cultured in medium containing 1 mg/ml G418 to select for successfully transfected cells. Clonal cell lines were subsequently generated for both the H_1_R and H_1_-YFP transfected cells. CHO cell lines were maintained in Dulbecco’s modified Eagles medium nutrient mix F12 (DMEM/F12) supplemented with 10% fetal calf serum and 2 mM L-glutamine at 37 °C in a humidified atmosphere of air/CO_2_ (19:1). HEK293T cells were maintained in Dulbecco’s modified Eagles medium (DMEM) supplemented with 10% fetal calf serum. HEK293T cells were grown in the presence of 1% penicillin/streptomycin prior to transient transfection for [^3^H]-mepyramine ligand binding studies.

### [^3^H]mepyramine saturation and competition binding

All [^3^H]mepyramine binding studies were performed on HEK293T cells transiently transfected to express the human H_1_R. This was achieved as follows, two million cells were seeded per 60 cm^2^ dish and transfected the following day with pcDEF3-HA-hH_1_R or pcDEF3-Nluc-hH_1_R using 5 μg DNA, 30 μg 25-kDa linear polyethylenimine in 150 mM NaCl per dish as described by Nijmeijer *et al*.^64^. Two days after transfection, cells were removed from the dish and washed in ice-cold phosphate buffered saline (PBS; 137 mM NaCl, 2.7 mM KCl, 10 mM Na_2_HPO_4_ and 2 mM KH_2_PO_4_). Cells pellets were obtained by centrifugation at 1900 × *g* for 10 min at 4 °C and stored at −20 °C until further experimentation. Cell pellets (expressing HA-hH_1_R) were reconstituted in HEPES buffered saline solution (HBSS; 145 mM NaCl, 5 mM KCl, 2 mM sodium pyruvate, 10 mM D-glucose, 10 mM HEPES, 1 mM MgSO_4_ and 1.3 mM CaCl_2_; pH = 7.4 at 37 °C) and dounce homogenized by plunging the pestle 10 times (Tamson, Bleiswijk, The Netherlands). For association binding experiments cells pellets (expressing either HA-hH_1_R or Nluc-hH_1_R) were instead reconstituted in phosphate buffer as described in Bosma *et al*.^65^ For saturation binding 1–4 μg of the cell homogenate was incubated (37 °C, 4 hours) with increasing concentrations of [^3^H]mepyramine (1–130 nM) both in the absence and presence of 10 µM mianserin to differentiate between total and non-specific binding. In competition binding experiments cell homogenate was incubated (37 °C, 4 hours) with 4 nM [^3^H]mepyramine and increasing concentrations competitor as required. Association binding experiments were performed according to the method described previously^65^, briefly, cell homogenate was incubation at 25 °C for between 1 and 41 min using 4 different concentrations of [^3^H]mepyramine (0–5 nM) both in the absence and presence of 10 µM mianserin to differentiate between total and non-specific binding. All ligand binding was performed under gentle agitation. Incubations were terminated by three rapid wash steps over GF/C filter plates using ice-cold 50 mM Tris-HCl buffer at pH7.4. Filter plates were dried at 52 °C before adding 25 μL Microscint-O per well. Finally, bound [^3^H]mepyramine was measured using a Wallac Microbeta counter (Perkin Elmer).

### Intracellular calcium mobilization assay

CHO H_1_R cells were grown to confluence in black-walled, clear-bottom 96-well plates. On the day of the experiment, media was replaced with 100 µl HBSS containing 2.5 mM probenecid, 2.3 µM Fluo-4AM (Life Technologies), 0.023% Pluronic F-127, 0.5 mM Brilliant Black BN and where required 100 nM of fluorescent antagonist. Cells were incubated in this Fluo-4 containing buffer for 45 min at 37 °C in the dark. Plates were then loaded onto a multi-well fluorometric imaging plate reader (FlexStation; Molecular Devices, Sunnyvale, CA) and Fluo-4 fluorescence was measured (excitation, 485 nm; emission, 520 nm) every 1.52 s for 200 s. HBSS or HBSS containing the required concentration of histamine was added after 15 s.

### Confocal Imaging

CHO H_1_R and CHO H_1_-YFP cells were grown to approximately 80% confluency on 8-well Labtek chambered coverglasses (Nunc Nalgene). On the day of the experiment, cells were washed twice in HBSS and then incubated in the presence or absence of 1 µM mepyramine for 30 min at 37 °C. Cells were then incubated with the required concentration of fluorescent ligand for 30 min prior to the collection of single equatorial confocal images. Images were obtained using a Zeiss LSM710 confocal microscope (Carl Zeiss GmbH, Jena, Germany) fitted with a 63× plan-Apochromat NA1.3 Ph3 oil-immersion lens within the School of Life Sciences Imaging Unit. For YFP, a 488 nm argon laser was used to excite the fluorophore and emission was detected using a BP505-30 filter. For the BY630 fluorophore, a 633 nm helium-neon laser was used for excitation and emission was detected using a 650 nm long pass filter. For all experiments a pinhole of 1 Airy Unit was used and fixed laser power, gain and offset for the BY630 containing compounds were kept the same for samples within each experiment.

### Cell Membrane Preparation for NanoBRET assays

HEK293T cell stably expressing Nluc-H_1_ were grown to confluence in 500 cm^2^ dishes. Normal growth media was replaced with ice-cold PBS and the cells were removed from the dish by scraping. The cells were then transferred to a 50 ml tube and centrifuged at 250 × g for 5 min. The supernatant was removed and the resulting pellets stored at −80 °C. Thawed pellets were resuspended in PBS and homogenized using an electrical homogenizer in 10 × 2 s bursts. Unbroken cells and nuclei were removed by centrifugation (1200 × g for 10 min) and the supernatant subsequently centrifuged at 41,415 g for 30 min. The membrane pellet was resuspended in PBS and homogenized by 20 passes of a glass-on-Teflon homogenizer. Protein concentration was determined using a BCA protein assay and membranes stored at −80 °C until required.

### NanoBRET binding assays

For whole cell NanoBRET assays, HEK293T cells stably expressing Nluc-H_1_ were seeded in white Thermo Scientific 96-well microplates and grown for 24 h prior to experimentation in normal growth medium. Immediately before experimentation, media was replaced with HBSS. For NanoBRET assay in membranes, membranes were diluted to the required concentration (10 µg/well) in binding buffer (50 mM Na_2_HPO_4_, 50 mM KH_2_PO_4_, pH 7.4) containing saponin (1 mg/ml) and placed in white Thermo Scientific 96-well microplates for 10 min prior to addition of compounds. For saturation and competition experiments in both whole cells and membranes the required concentrations of fluorescent ligand and competing ligand were added simultaneously. Plates were then incubated for 1 h at 37 °C (no CO_2_). After 1 h, 10 µM furimazine (Promega) was added to each well and fluorescence and luminescence measured after 5 min. For association and competition association kinetic experiments, 10 µM furimazine was added to each well and incubated at room temperature in the dark for 15 min to allow the luminescence signal to reach equilibrium. For association kinetics experiments, the required concentration of fluorescent ligand in the presence or absence of 10 µM doxepin was added simultaneously. For competition association kinetic experiments, 10 nM **10** was added in the presence or absence of the indicated concentration of unlabelled ligand. Non-specific binding was determined by the addition of 10 nM **10** and 10 µM doxepin. The plates were then read immediately with each well being read once per minute for 60 min. For all experiments fluorescence and luminescence was read sequentially using a PHERAstar FS plate reader (BMG Labtech) at room temperature. Filtered light emissions were measured at 460 nm (80-nm bandpass) and at >610 nm (longpass) and the raw BRET ratio was calculated by dividing the >610-nm emission by the 460-nm emission.

### Data analysis

All data were analysed and presented using Prism 6 (GraphPad Software, San Diego, CA).

Total and non-specific saturation binding curves were fitted simultaneously using equation (1):

1 $$dpm=\frac{{B}_{max}\times [B]}{[B]+{K}_{D}}+((M\,\times [B])+C)$$

where B_max_ is the maximal response, [B] is the concentration of fluorescent ligand in nM, K_D_ is the equilibrium dissociation constant in nM, M is the slope of the non-specific binding component and C is the intercept with the Y-axis.

We fitted the both the radioligand and fluorescence competition binding curves using equation (2):

2 $${{\boldsymbol{K}}}_{{\boldsymbol{i}}}=\frac{{\boldsymbol{I}}{{\boldsymbol{C}}}_{50}}{1+\frac{[{\boldsymbol{L}}]}{{{\boldsymbol{K}}}_{{\boldsymbol{D}}}}}$$

where [L] is the concentration of [^3^H]mepyramine, **10** or **23** in nM and K_D_ is the equilibrium dissociation constant of the labelled ligand in nM (24 nM for [^3^H]mepyramine, 9.2 nM for **10** in whole cells, 28.8 nM for **10** in membranes and 9.8 nM for **23**). The IC_50_ is calculated as in equation (3):

3 $$ \% \,{inhibition}\,{of}\,{specific}\,{binding}=\frac{100\times [A]}{[A]+I{C}_{50}}$$

where [A] is the concentration of unlabelled competing drug and IC_50_ is the molar concentration of this competing ligand required to inhibit 50% of the specific binding of the concentration [L] of the labelled ligand.

For the calcium mobilization experiments, estimated affinity values (pK_B_) were calculated from the shift of the agonist concentration response curves in the presence of the fluorescent antagonists using equation (4):

4 $$DR=1+\frac{[B]}{{K}_{B}}$$

Where DR (dose ratio) is the ratio of the agonist concentration required to stimulate an identical response in the presence and absence of antagonist, [B]. As there was a decrease in observed maximal efficacy of histamine in the presence of all six fluorescent antagonists, the EC_25_ value was used to determine the DR^39^.

From association kinetic data, non-specific binding was determined for each concentration of fluorescent ligand at each time point by the addition of 10 µM doxepin and was subtracted from total binding. Then k_on_, k_off_ and K_D_ values were obtained from the data using equation (5):

5 $${{\boldsymbol{K}}}_{{\boldsymbol{D}}}=\frac{{{\boldsymbol{k}}}_{{\boldsymbol{off}}}}{{{\boldsymbol{k}}}_{{\boldsymbol{on}}}}$$

Where K_D_ is the equilibrium dissociation constant and k_off_ is the dissociation rate constant of the ligand in min^−1^. k_on_ is the association rate constant in M^−1^ min^−1^ and is calculated as follows in equation (6):

6 $${k}_{on}=\frac{{k}_{obs}-{k}_{off}}{[L]}$$

Where [L] is the ligand concentration in M and k_obs_ is calculated from global fitting of the data to the following monoexponential association function (equation (7)):

7 $$Y={Y}_{max}(1-{e}^{-{k}_{obs}t})$$

Here Y_max_ equals levels of binding at infinite time (t), and k_obs_ is the rate constant for the observed rate of association.

The binding kinetics of unlabelled ligands was quantified in a competition association assay based on the theoretical framework proposed by Motulsky and Mahan^42^. The k_on_ and k_off_ of the unlabelled ligands were obtained using equations (8–14):

8 $${K}_{A}={k}_{1}[L].\,{10}^{-9}+{k}_{2}$$

9 $${K}_{B}={k}_{3}[I].\,{10}^{-9}+{k}_{4}$$

10 $$S=\sqrt{{({K}_{A}-{K}_{B})}^{2}+4.\,{k}_{1}}.{k}_{3}.L.I.\,{10}^{-18}$$

11 $${K}_{F}=0.5({K}_{A}+{K}_{B}+S)$$

12 $${K}_{s}=0.5({K}_{A}+{K}_{B}-S)$$

13 $$Q=\frac{{B}_{max}.{k}_{1}.L.\,{10}^{-9}}{{K}_{F}-{K}_{S}}$$

14 $$Y=Q.(\frac{{k}_{4}.({K}_{F}-{K}_{S})}{{K}_{F}-{K}_{S}}+\frac{{k}_{4}-{K}_{F}}{{K}_{F}}{e}^{(-{K}_{F}.X)}-\frac{{k}_{4}-{K}_{S}}{{K}_{S}}{e}^{(-{K}_{S}.X)})$$

Where k_1_ is the k_on_ of the labelled ligand (M^−1^min^−1^), k_2_ is the k_off_ of the labelled ligand (min^−1^), L is the concentration of the labelled ligand in nM, I is the concentration of the unlabelled competitior in nM, X is the time (min) and Y is the specific binding of the labelled ligand (NanoBRET ratio). From these equations the k_on_ (k_3_, M^−1^min^−1^), k_off_ (k_4_, min^−1^) of the unlabelled ligand and the B_max_ (total binding given as maximal BRET ratio) were calculated.

Statistical significance was determined by Student’s unpaired *t* test where p < 0.05 was deemed to be statistically significant throughout this study.

### Data availability

The datasets generated and analysed during the current study are available from the corresponding author on reasonable request.

## Electronic supplementary material

Supplementary Information
