# Supplementary material for: Development of novel fluorescent histamine H1-receptor antagonists to study ligand-binding kinetics in living cells
Source: Sci Rep. 2018 Jan 25;8:1572. doi: 10.1038/s41598-018-19714-2 (PMC5785503; doi:10.1038/s41598-018-19714-2)
Supplement: Supplementary file 1 — Supplementary Information [file 41598_2018_19714_MOESM1_ESM.pdf]

Supplemental Information for

Development of novel fluorescent histamine H_1-receptor antagonists to study ligand-binding kinetics in living cells.

*Leigh A Stoddart[1,4], Andrea J Vernall[2], Monica Bouzo-Lorenzo,[1,4], Reggie Bosma[3], Albert J Kooistra[3], Chris de Graaf[3], Henry F Vischer[3], Rob Leurs[3], Stephen J Briddon[1,4], Barrie Kellam[*2,4], Stephen J Hill[*1,4]*

[1] Division of Pharmacology, Physiology and Neuroscience, School of Life Sciences, University of Nottingham, Nottingham, NG7 2UH, UK.

[2] School of Pharmacy, Division of Biomolecular Science and Medicinal Chemistry, Centre for Biomolecular Sciences, University of Nottingham, Nottingham, NG7 2RD, UK.

[3] Department of Chemistry and Pharmaceutical Sciences, Division of Medicinal Chemistry, Amsterdam Institute for Molecules, Medicines and Systems (AIMMS), Vrije Universiteit, Amsterdam, PO Box 7161, Amsterdam, The Netherlands.

[4] Centre of Membrane Proteins and Receptors, University of Birmingham and University of Nottingham, The Midlands, U.K.

*Corresponding authors: Stephen J Hill, steve.hill@nottingham.ac.uk; Barrie Kellam, barrie.kellam@nottingham.ac.uk

Figure S1. Scheme for the synthesis of mepyramine and VUF13816 based fluorescent ligands.

(a) Fluorescent mepyramine analogues.

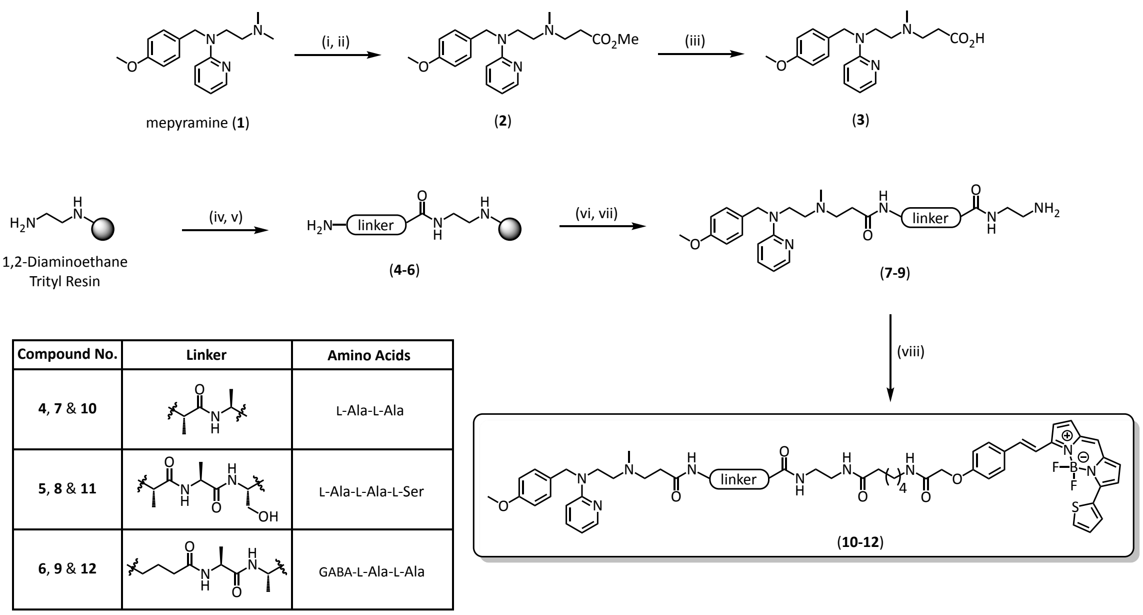

Reagents and conditions: (i) 1-Chloroethyl chloroformate, 1,2-dichloroethane, $NaHCO_3$, then refluxing MeOH, 7%. (ii) Methyl acrylate, 1,2-dichloroethane, 85%. (iii) 0.2M LiOH aq, THF, 90%. (iv) Fmoc-NH-amino acid-CO_2H, HBTU DIPEA, DMF. (v) 20% Piperidine/DMF; repeat steps (iv) and (v) until desired peptide sequence assembled. (vi) **3**, HATU, DIPEA, DMF. (vii) TFA/DCM (refer to following section for product quantities isolated). (viii) BODIPY 630/650-X-SE, DMF, 37 - 64% yield following RP-HPLC purification.

(b) Fluorescent VUF13816 analogues.

.

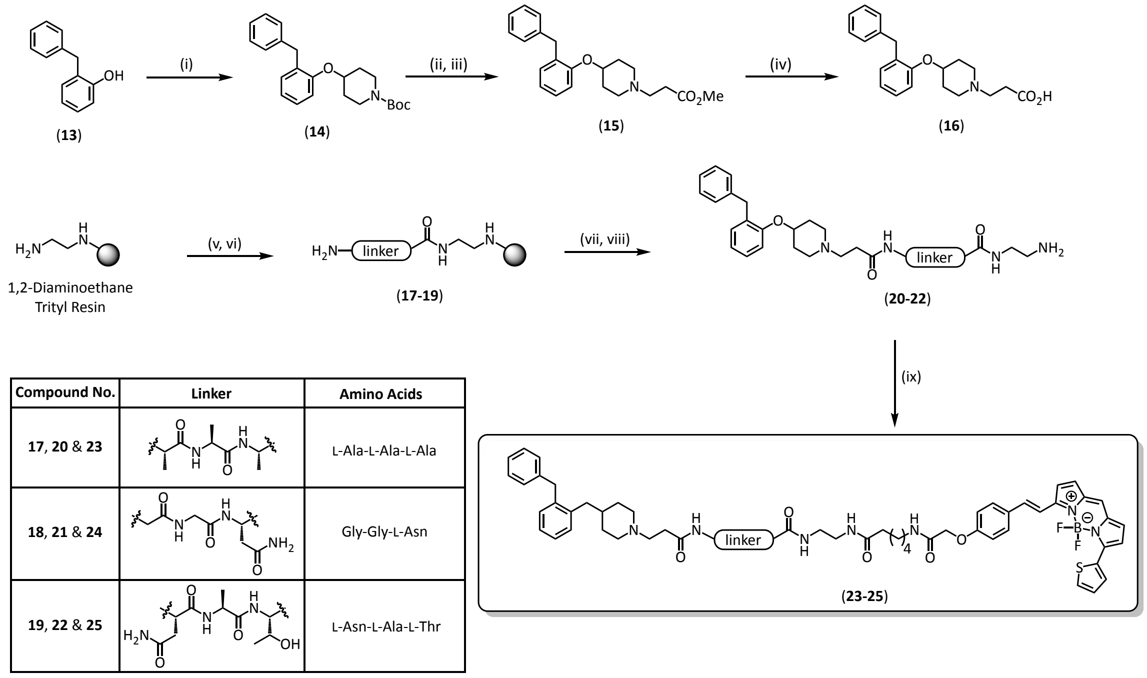

Reagents and conditions: (i) *N-tert*-butoxycarbonyl -4-bromopiperidine, KOH, MeOH, reflux, 28%. (ii) Trifluoroacetic acid, DCM, quantitative. (iii) Methyl acrylate, 1,2-dichloroethane, 89%. (iv) 0.2M LiOH aq, THF, 90%. (v) Fmoc-NH-amino acid-CO_2H, HBTU DIPEA, DMF. (vi) 20% Piperidine/DMF; repeat steps (v) and (vi) until desired peptide sequence assembled. (vii) **16**, HATU, DIPEA, DMF. (viii) TFA/DCM (refer to following section for product quantities isolated). (ix) BODIPY 630/650-X-SE, DMF, 73 - 73% yield after RP-HPLC purification.

Figure S2. Live cell confocal imaging of the H_1R in CHO cells using mepyramine-based fluorescent ligands.

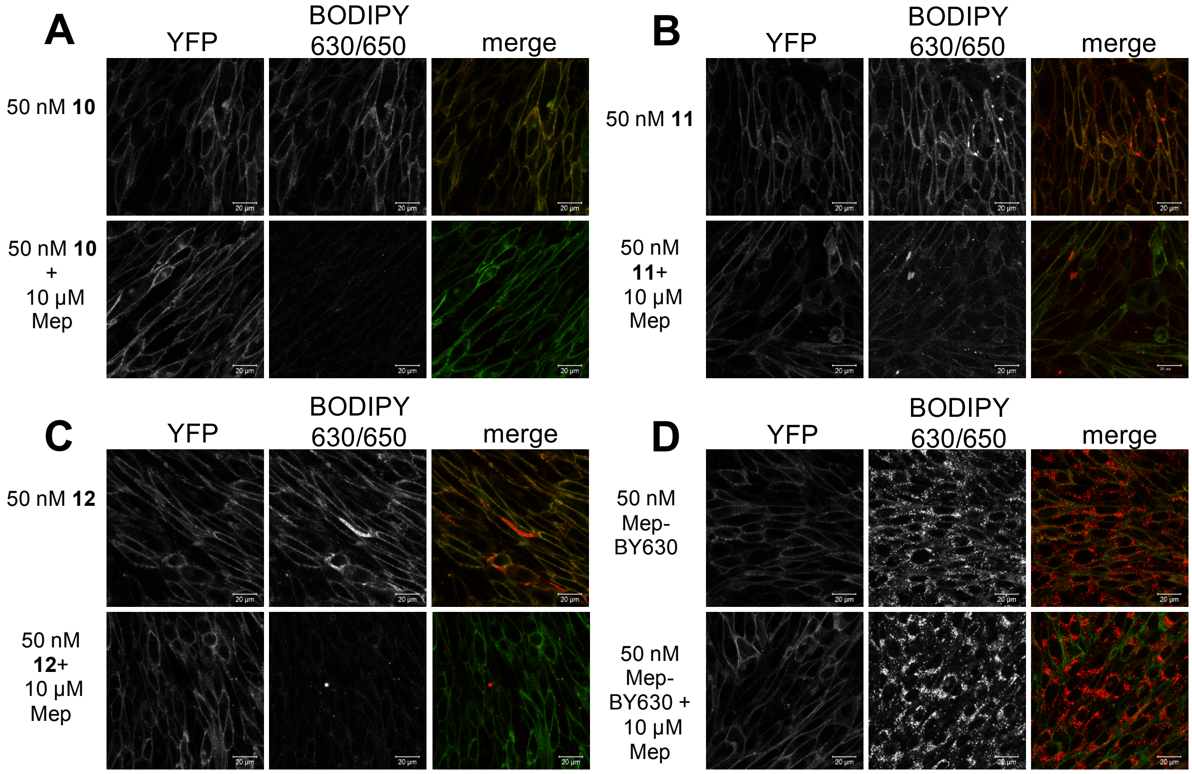

Live cell confocal images of CHO cells expressing H_1-YFP and incubated with 50 nM (A) **10**, (B) **11**, (C) **12** and (D) mepyramine-X-BODIPY630-650 (Mep-X-BY630; Rose et al., 2012) at 37°C in the absence (upper panels) or presence of 10 µM mepyramine (Mep; lower panels). Single equatorial images were taken of YFP (left hand panels) and BY630/650 (right hand panels). YFP and BY630/650 images are shown in greyscale to avoid issues with colour rendering. Images in the right hand panels represent the merge of the YFP and BY630/650 images. For each compound, images in the presence and absence of mepyramine were obtained using identical settings for laser power, detector offset and gain. Data shown are representative of images obtained in three independent experiments. Scale bars = 20 µm.

Figure S3. Live cell confocal imaging of the H_1R in CHO cells using VUF13816 based fluorescent ligands.

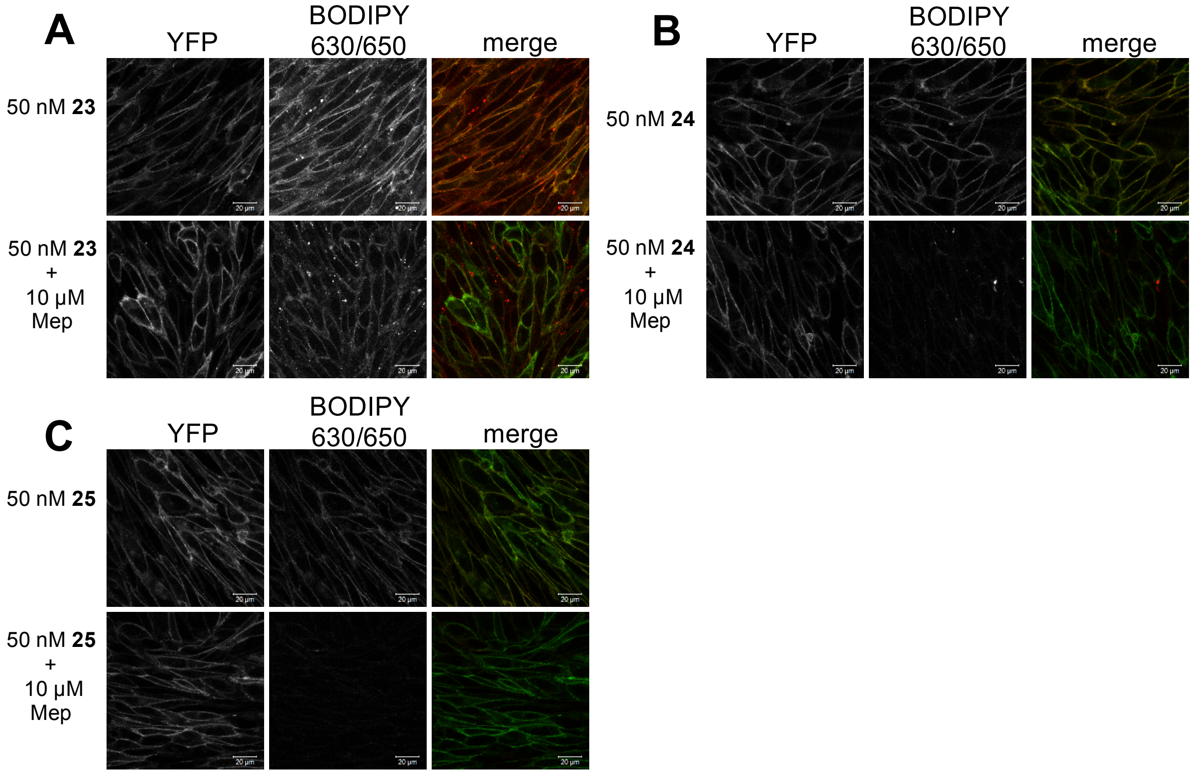

Live cell confocal images of CHO cells expressing H_1-YFP and incubated with 50 nM (A) **23**, (B) **24** and (C) **25** at 37°C in the absence (top panels) or presence of 10 µM mepyramine (Mep) (bottom panels). Single equatorial images were taken of YFP (left hand panels) and BY630/650 (right hand panels). YFP and BY630/650 images are shown in greyscale to avoid issues with colour rendering. Images in the right hand panels represent the merge of the YFP and BY630/650 images. For each compound, images in the presence and absence of mepyramine were obtained using identical settings for laser power, detector offset and gain. Data shown are representative of images obtained in three independent experiments. Scale bars = 20 µm.

Figure S4. NanoBRET saturation studies in whole cells with mepyramine and VUF13816 based fluorescent ligands

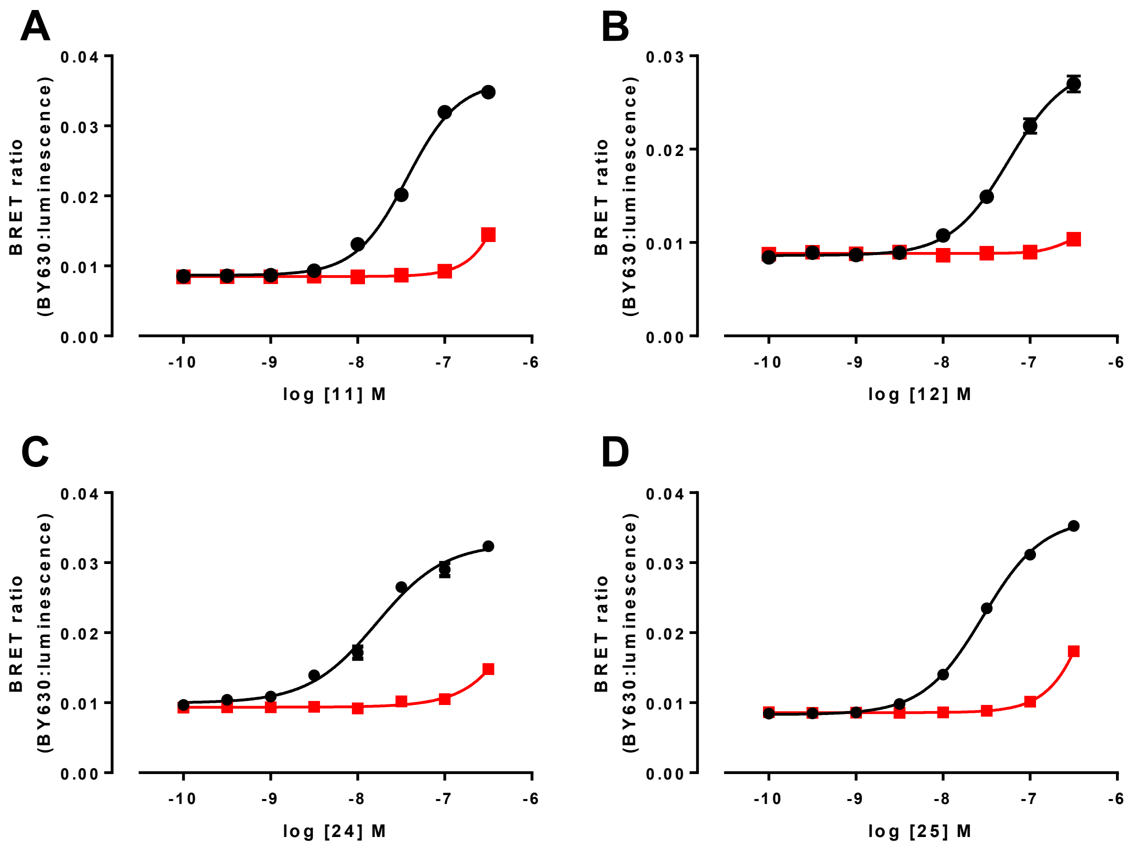

Saturation BRET binding curves from Nluc-H$_1$R HEK293T cells treated with increasing concentrations of **11** (A), **12** (B), **24** (C) and **25** (D) in absence (black circles) or presence (red squares) of 10μM mepyramine. The data shown are representative of four (A), five (B, E and F) independent experiments performed in triplicate.

Figure S5. Stability of NanoBRET signal with Nluc-H_1 and compound 10 over time.

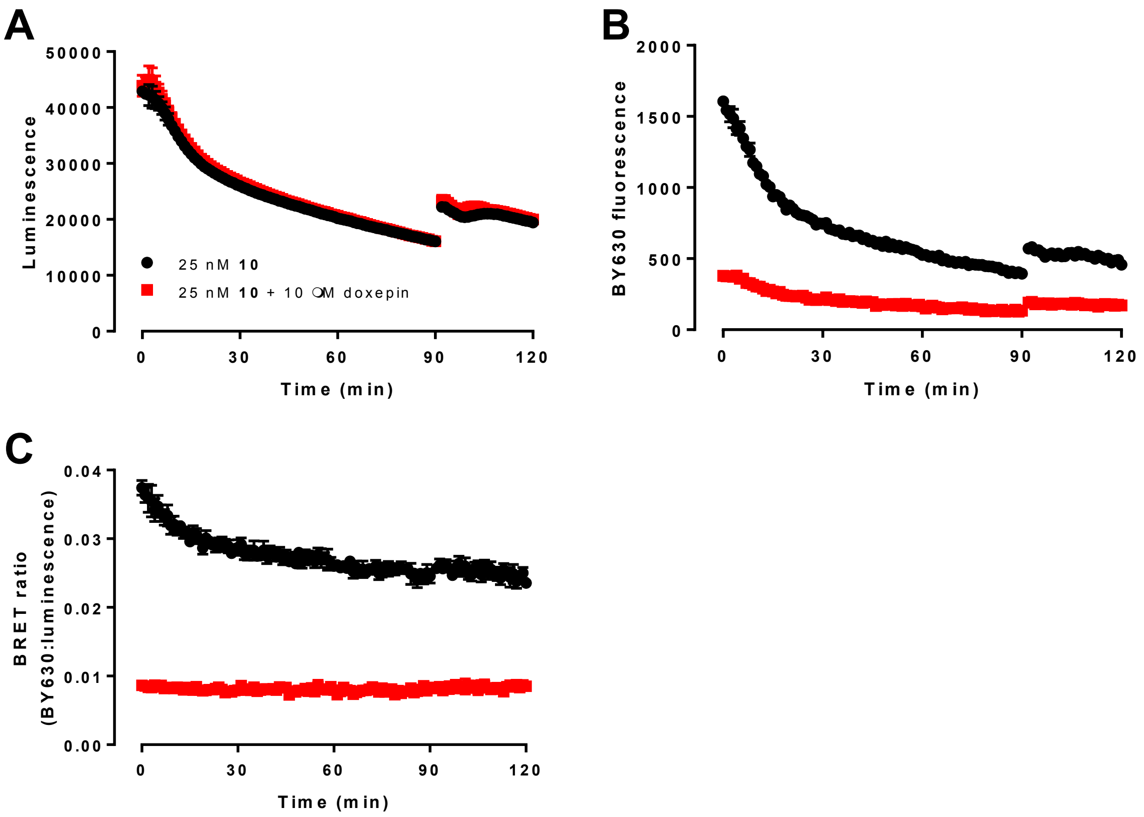

Luminescence (A), fluorescence (B) and BRET ratio (C) signal from Nluc-H_1R HEK293T cells stably expressing treated with 25 nM **10** in absence or presence of 10μM doxepin. Following the addition of furimazine (0.5μM), the luminescence and the fluorescent signals were measured every min for 90 min at room temperature. After this 90 min another dose of furimazine (0.5μM) was added and the luminescence and fluorescence were measured every min for a further 30 min at room temperature. The data shown are representative of four independent experiments performed in triplicate.

Figure S6. Effect of the addition of saponin to cell membranes in the BRET signal.

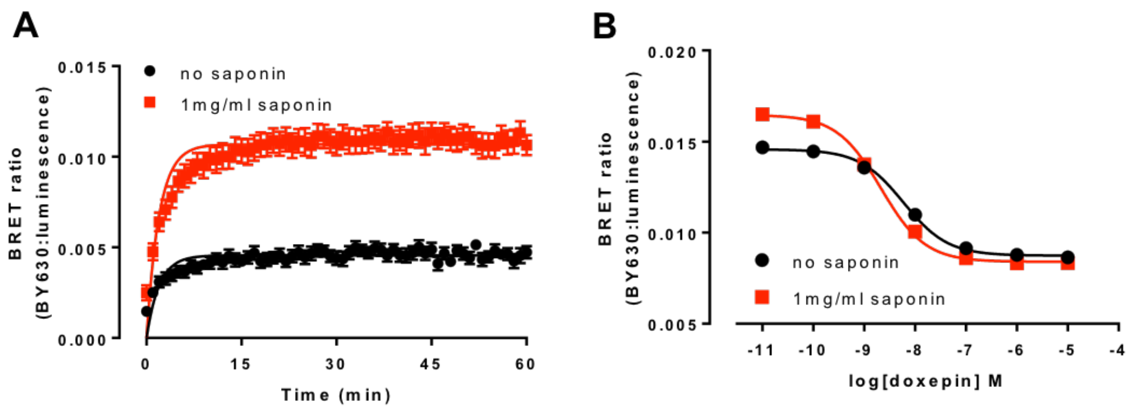

A. Membranes of Nluc-H_1R HEK293T cells treated with 25 nM **10** in absence (black circles) or presence (red squares) of 1mg/mL of saponin were monitored by BRET at room temperature every min for 60 min. **B.** Inhibition of BRET signal in membranes of Nluc-H_1R HEK293T cells treated with 25 nM **10** and increasing concentrations of doxepin in absence (black circles) or presence (red squares) of 1mg/mL of saponin. The data shown are representative of four independent experiments performed in triplicate.
